# Genes *ycfR*, *sirA* and *yigG* Contribute to the Surface Attachment of *Salmonella enterica* Typhimurium and Saintpaul to Fresh Produce

**DOI:** 10.1371/journal.pone.0057272

**Published:** 2013-02-22

**Authors:** Joelle K. Salazar, Kaiping Deng, Mary Lou Tortorello, Maria T. Brandl, Hui Wang, Wei Zhang

**Affiliations:** 1 Institute for Food Safety and Health, Illinois Institute of Technology, Bedford Park, Illinois, United States of America; 2 United States Food and Drug Administration, Bedford Park, Illinois, United States of America; 3 Produce Safety and Microbiology Research Unit, Western Regional Research Center, Agricultural Research Service, United States Department of Agriculture, Albany, California, United States of America; 4 Food Safety Research Center, Shanghai Institute for Biological Sciences, Chinese Academy of Sciences, Shanghai, China; Indian Institute of Science, India

## Abstract

*Salmonella enterica* is a frequent contaminant of minimally-processed fresh produce linked to major foodborne disease outbreaks. The molecular mechanisms underlying the association of this enteric pathogen with fresh produce remain largely unexplored. In our recent study, we showed that the expression of a putative stress regulatory gene, *ycfR*, was significantly induced in *S. enterica* upon exposure to chlorine treatment, a common industrial practice for washing and decontaminating fresh produce during minimal processing. Two additional genes, *sirA* involved in *S. enterica* biofilm formation and *yigG* of unknown function, were also found to be differentially regulated under chlorine stress. To further characterize the roles of *ycfR*, *sirA*, and *yigG* in *S. enterica* attachment and survival on fresh produce, we constructed in-frame deletions of all three genes in two different *S. enterica* serovars, Typhimurium and Saintpaul, which have been implicated in previous disease outbreaks linked to fresh produce. Bacterial attachment to glass and polystyrene microtiter plates, cell aggregation and hydrophobicity, chlorine resistance, and surface attachment to intact spinach leaf and grape tomato were compared among wild-type strains, single-gene deletion mutants, and their respective complementation mutants. The results showed that deletions of *ycfR, sirA,* and *yigG* reduced bacterial attachment to glass and polystyrene as well as fresh produce surface with or without chlorine treatment in both Typhimurium and Saintpaul. Deletion of *ycfR* in Typhimurium significantly reduced bacterial chlorine resistance and the attachment to the plant surfaces after chlorinated water washes. Deletions of *ycfR* in Typhimurium and *yigG* in Saintpaul resulted in significant increase in cell aggregation. Our findings suggest that *ycfR*, *sirA*, and *yigG* collectively contribute to *S. enterica* surface attachment and survival during post-harvest minimal processing of fresh produce.

## Introduction

Salmonellosis, a human infectious disease caused by *Salmonella enterica*, is the leading cause of bacterial foodborne illnesses (17.4 out of 100,000 persons), hospitalizations (54%) and deaths (43%) in the U. S. [1], [2]. Infections of salmonellosis have mainly been traced back to consumption of products of animal origin; however, an increasing concern is directed to the *S. enterica* outbreaks associated with fresh produce [3] which poses a significant threat to food safety and public health due to the growing consumption of minimally processed fruits and vegetables as part of a healthy diet [4].

*S. enterica* can rapidly adapt to environmental stresses and survive for long periods of time in various non-host habitats including agricultural fields and the surface of fresh produce [5]. *S. enterica* has been implicated in several recent multistate outbreaks linked to contaminated fruits and vegetables including lettuce (*S.* Typhimurium and *S.* Braenderup), alfalfa sprouts (*S.* Enteritidis and *S.* Saintpaul), jalapeño peppers (*S.* Saintpaul), tomatoes (*S.* Saintpaul and *S.* Typhimurium), papaya (*S*. Agona) and cantaloupe (*S.* Saintpaul and *S*. Panama) [1], [2], [5]–[12].

Among many different *S. enterica* serovars, we selected to analyze Typhimurium and Saintpaul in this study because these two serovars have been implicated in major human foodborne outbreaks of salmonellosis linked to fresh produce and related products such as tomatoes, alfalfa sprouts, and orange juice [2], [5], [6], [9], [13], [14]. According to the U. S. Centers for Disease Control and Prevention, *S.* Typhimurium and *S.* Saintpaul accounted for 17% and 2.2% of all produce related human salmonellosis in 2009, respectively [1], [2].

Current industrial practice to decontaminate fresh produce harvested directly from the field involves the use of wash water supplemented with low concentrations of sodium hypochlorite (or chlorine). Chlorine is an oxidative agent that is used to reduce the overall bacterial load on produce surface and prevent cross-contamination in the wash water during minimal processing. However, studies have shown that *S. enterica* is capable of attaching to the produce surface and forming protective layers of biofilms [15], making it difficult to completely inactivate the attached pathogens by chlorinated water washes alone.

The genome of *S. enterica* contains over 4,000 protein-coding genes; some of which have been shown to play roles in bacterial stress resistance, motility, biofilm production, and virulence [16]. Among these genes, *ycfR* (258-bp) is a highly conserved gene in many Gram-negative bacterial species which is known to play a role in stress resistance and biofilm production in *E. coli* by altering cell surface hydrophobicity [17]. Our recent studies of *ycfR* in *E. coli* O157:H7, *S.* Typhimurium, and *S.* Enteritidis showed that this gene was mostly up-regulated under chlorine stress, suggesting a potential role in bacterial chlorine resistance [18]–[20]. Two additional genes, *sirA* (111-bp) and *yigG* (459-bp), were selected for analysis in this study because these two genes were also shown to be differentially regulated under chlorine stress and potentially involved in bacterial biofilm production, gene regulation, and virulence in *S. enterica* [20].

The major objective of this current study was to investigate the functional roles of *ycfR*, *sirA*, and *yigG* in the attachment and survival of *S. enterica* on fresh produce during procedures related to post-harvest minimal processing. We constructed in-frame deletion and complementation mutants for all three genes in both *S.* Typhimurium and *S.* Saintpaul as listed in Table 1. We compared the relative abilities of these mutants with their respective wild-type strains for chlorine resistance, biofilm production, cell aggregation and hydrophobicity, as well as surface attachment to fresh spinach leaves and grape tomatoes. Results from this study may improve our basic understanding of the molecular mechanisms that enable *S. enterica* to attach to produce and survive post-harvest decontamination processes.

**Table 1:** Bacterial strains and plasmids used in this study.

Strain or plasmid	Designation	Reference
*S.* Typhimurium LT2	Wild-type	(22)
*S.* Saintpaul	99A3746	CSDH^a^
*S.* Typhimurium LT2 *ΔycfR::cat*		This study
*S.* Typhimurium LT2 *ΔyigG::cat*		This study
*S.* Typhimurium LT2 *ΔsirA::cat*		This study
*S.* Saintpaul *ΔycfR::cat*		This study
*S.* Saintpaul *ΔyigG::cat*		This study
*S.* Saintpaul *ΔsirA::cat*		This study
*S.* Typhimurium LT2 *ΔycfR::cat* (pJS-8)		This study
*S.* Typhimurium LT2 *ΔyigG::cat* (pJS-16)		This study
*S.* Typhimurium LT2 *ΔsirA::cat* (pJS-18)		This study
*S.* Saintpaul *ΔycfR::cat* (pJS-10)		This study
*S.* Saintpaul *ΔyigG::cat* (pJS-16)		This study
*S.* Saintpaul *ΔsirA::cat* (pJS-18)		This study
*E. coli* BW25113 (pKD46)		(12)
*E. coli* BW25141 (pKD3)		(12)
pACYC177		NEB^b^

a California State Department of Health.

b New England BioLabs.

## Results

### Gene Annotation and Phylogenetic Analysis

**Figure 1:**
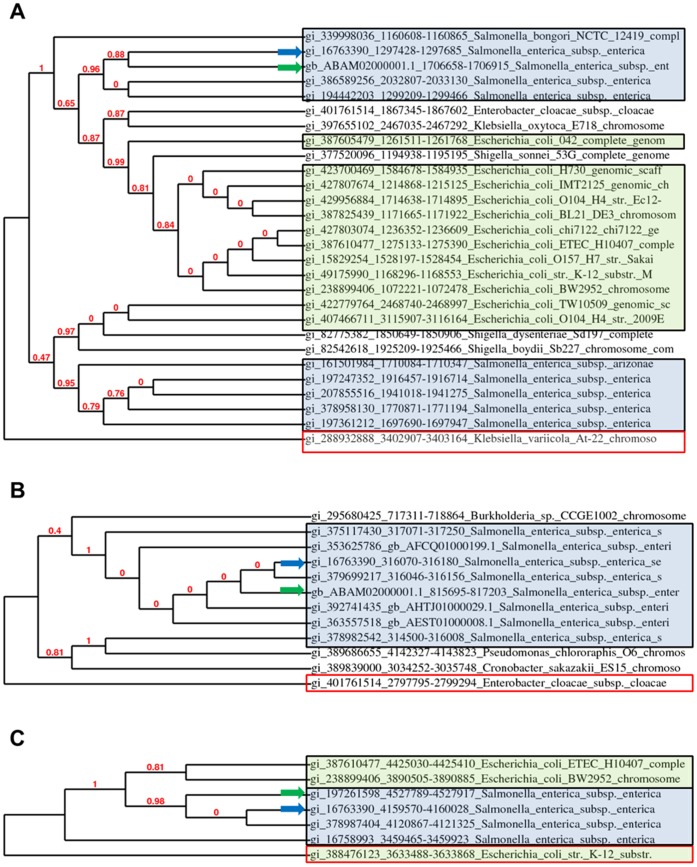
Phylogenetic relatedness of *ycfR*, *sirA*, and *yigG* in *S. enterica* and their homologs in related bacterial species. Cladograms for *ycfR* (A), *sirA* (B), and *yigG* (C) with branch support values displayed at nodes were reconstructed using PhyML 3.0 aLRT and TreeDyn 198.3. (http://www.phylogeny.fr/) (22–28). *Salmonella* sp. and *E. coli* sp. are indicated by blue and green boxes, respectively. An outgroup is indicated with a red box. Blue and green arrows indicate locations of *S.* Typhimurium and *S.* Saintpaul, respectively, in the cladograms.

YcfR is a multiple stress resistance protein and biofilm regulator in *E. coli* K-12, which also plays a role in the chlorine resistance of *E. coli* O157:H7 [18]. However, the function of *ycfR* in *S. enterica* has not been previously reported. BLAST search and sequence alignment of *ycfR* in *S. enterica* and its homologs in other Gram-negative bacteria showed that the DNA sequences of this gene display some polymorphisms; however, the amino acid sequences are identical among various *S. enterica* serovars including Typhi, Saintpaul, Newport, and Montevideo (Fig. S1A). Gene *ycfR* is also completely conserved in the fully sequenced *E. coli* K-12 and O157:H7 genomes. BhsA, a multiple stress resistance protein involved in biofilm formation and hydrophobicity, is a homolog of YcfR in *Shigella dysenteriae* and *Klebsiella oxytoca* [17], [21]. Fig. 1A is a cladogram that shows the phylogenetic relatedness of *ycfR* in *S*. *enterica* and its homologs in other bacterial species or subspecies including *S. bongori*, *E. coli*, *S. dysenteriae* and *K. oxytoca* [22]–[28].

Gene *sirA* encodes a response regulator Invasol SirA [16]. A BLASTp search showed that SirA in *S*. Typhimurium shares 100% amino acid sequence identity with its homologs in other *S. enterica* serovars including Minnesota, Newport, Dublin, and Saintpaul. SirA homologs in other bacterial species include EvpB in *Burkholderia* sp., ImpC in *Enterobacter cloacae*, TssC1 in *Pseudomonas chlororaphis*, and EvpB in *Cronobacter sakazakii* [29], [30]. Gene *yigG* encodes a putative inner membrane protein with unknown function and is 100% identical between Typhimurium, Saintpaul, and Typhi. A homolog with 82% amino acid sequence identity was also found in *E. coli* K-12 and BW2952 genomes. Cladograms of *sirA* and *yigG* are shown in Fig. 1B and 1C, respectively [22]–[28]. Similar to *ycfR*, these two genes also display some sequence polymorphisms among different Gram-negative bacteria (Fig. S1B and S1C). Interestingly, both *sirA* and *yigG* are absent in *S.* Enteritidis, a prevalent *S. enterica* serotype that causes most foodborne salmonellosis in the U. S. [20].

### Chlorine Resistance

**Figure 2:**
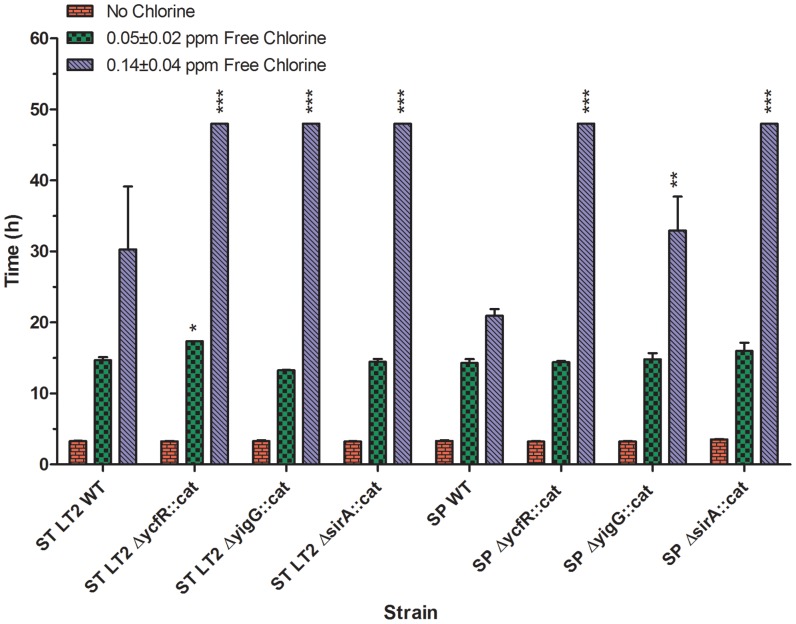
Extended lag phase of *S. enterica* wild-types and mutants in BIH broth with and without chlorine stress. Standard deviations represent three independent experiments. Significant differences in comparison to the parent strains under the same experimental condition are shown as *(*P*<0.5), **(*P*<0.001), and ***(*P*<0.0001).

*ycfR* deletion mutants in *S.* Typhimurium and *S.* Saintpaul were compared for their relative resistance to sublethal chlorine stress (i.e. 0.05±0.02 ppm sodium hypochlorite in BHI broth) for 48 h at 37°C in an automated Bioscreen C system. Chlorine resistance was determined based on the extended lag phase, which was defined as the time period (h) between initial inoculation and the time when bacterial OD_600_ reached 0.2 as previously described [18]. Figure 2 shows that all *S.* Typhimurium and *S.* Saintpaul wild-types and mutants had an approximate 3 h lag phase in BHI broth without chlorine. When subjected to 0.05±0.02 ppm free chlorine, *ΔycfR::cat* in *S.* Typhimurium displayed the longest lag phase (approximately 17.4 h), significantly different from other mutants (*P*<0.05). *ΔycfR::cat* in *S.* Saintpaul showed a similar lag phase as its parent strain. This suggests that deletion of *ycfR* led to greater chlorine sensitivity in *S.* Typhimurium but not in *S*. Saintpaul. When the free chlorine concentration was increased to 0.14±0.04 ppm, *S.* Typhimurium and *S.* Saintpaul wild-types were still able to grow but with significantly extended lag phases at *P*<0.001 and *P*<0.0001, respectively. However, both *ΔycfR::cat* mutants did not grow after 48 h of incubation.

To determine if *sirA* and *yigG* also contribute to the chlorine resistance of *S. enterica*, isogenic *ΔsirA* and *ΔyigG* mutants in *S.* Typhimurium and *S.* Saintpaul were constructed and subjected to low sublethal chlorine stress (0.05±0.02 ppm free chlorine). All *ΔsirA::cat* and *ΔyigG::cat* mutants in *S.* Typhimurium and *S.* Saintpaul displayed similar lag phases to those of wild types (Fig. 2). However, when subjected to a higher concentration of chlorine (0.14±0.04 ppm free chlorine), all mutants except for *ΔyigG::cat* in *S.* Saintpaul were unable to grow, indicating that both *sirA* and *yigG* are required at least for *S.* Typhimurium to grow under chlorine stress.

### Cell Aggregation and Hydrophobicity

**Figure 3:**
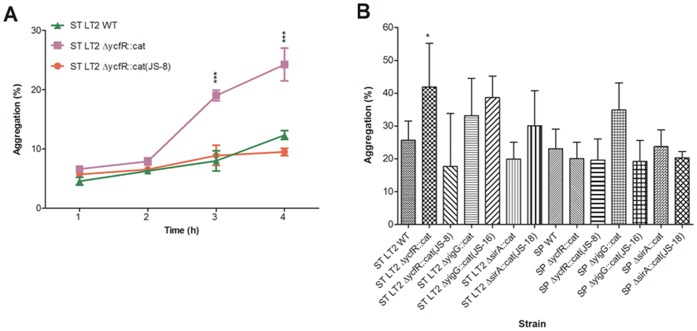
*S. enterica* aggregation assays. A) Aggregation of *S.* Typhimurium LT2 WT, *ΔycfR::cat* mutant, and *ΔycfR::cat* (JS-8) complement over 4 h. B) Aggregation of all *S. enterica* strains after 24 h incubation at room temperature. Standard deviations represent three independent experiments. Significant differences in comparison to the parent strains under the same condition are shown as *(*P*<0.5), **(*P*<0.001), and ***(*P*<0.0001).

To test whether deletion of *ycfR* in *S. enterica* may cause changes to cell surface properties such as aggregation and hydrophobicity as previously reported in its close relative *E. coli* O157:H7 [18], *S. enterica* wild-type strains, *ΔycfR::cat* and complementation mutants were compared for these phenotypes. In the cell aggregation assay, *S.* Typhimurium wild-type and the *ycfR* complement mutant settled moderately whereas the *ΔycfR::cat* in *S.* Typhimurium aggregated quickly with significant differences (*P*<0.0001) at 3 h and 4 h (Fig. 3A). After 24 h, significant differences (*P*<0.05) were detected between *ΔycfR::cat* in *S.* Typhimurium and its parent strain (Fig. 3B). Interestingly, no change in aggregation was seen with *ΔycfR::cat* in *S*. Saintpaul, indicating that deletion of *ycfR* leads to greater aggregation in *S.* Typhimurium but not in *S.* Saintpaul. Assays were also conducted to test the hydrophobicity of the outer membrane of the bacterium. No remarkable difference in hydrophobicity was noted among the wild-types and mutants (data not shown), suggesting that, unlike in *E. coli*, *ycfR* may not be involved in hydrophobicity in *S. enterica*. In addition, hydrophobicity and aggregation (Fig. 3B) of the *ΔsirA::cat* and *ΔyigG::cat* mutants were assayed. No significant change of hydrophobicity and aggregation was evident for the mutants.

### Biofilm Production

**Figure 4:**
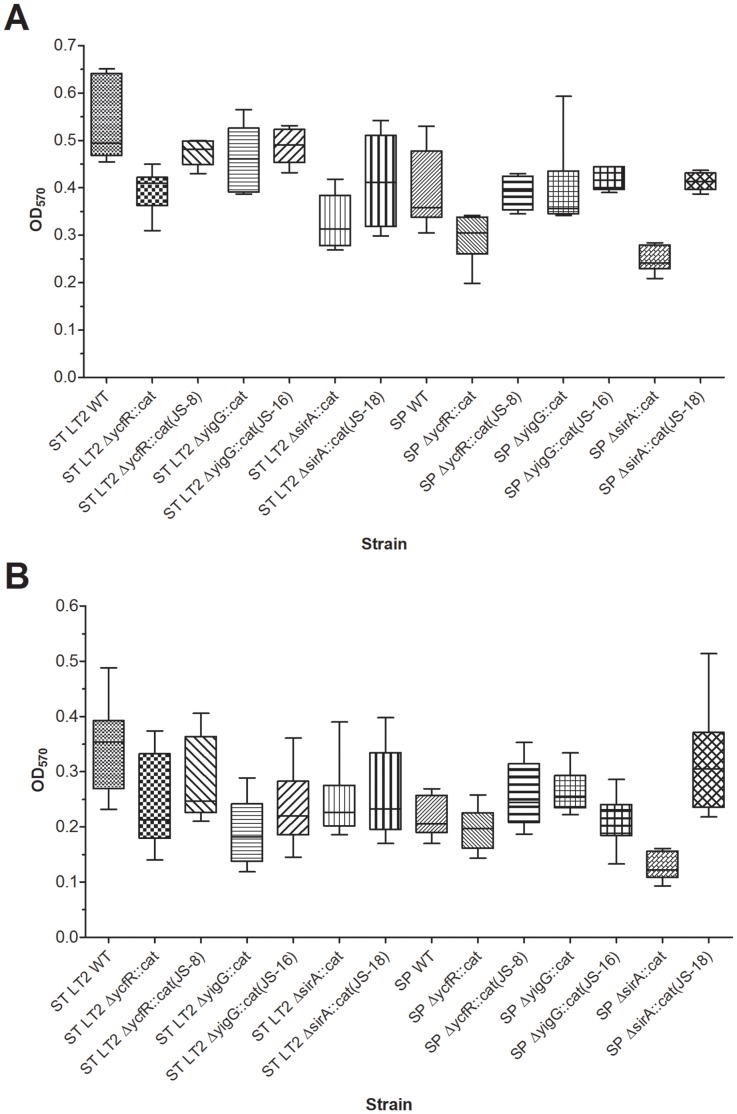
*S. enterica* crystal violet attachment assays. The bacterial attachment is quantified by OD_570_ readings. A) Quantification of bacterial attachment in glass test tubes. B) Quantification of bacterial attachment in polystyrene 96-well plates. All assays were conducted for 24 h at 37°C. Middle horizontal line in each box represents the median of the entire data set; the upper and lower horizontal lines represent the upper quadrant median and the lower quadrant median, respectively. Detailed data analysis is given in Table 2.

Crystal violet assays were performed to determine if *ycfR* contributes to biofilm formation in *S. enterica*, as previously reported in *E. coli* O157:H7 [18]. All the tested strains and mutants attached better on glass (Fig. 4A) than on polystyrene surfaces (Fig. 4B) when incubated in BHI broth at 37°C. *S.* Typhimurium showed a significant difference in attachment to glass surface between the wild-type and *ΔycfR::cat* mutant (*P* = 0.006) whereas the differences between the *S.* Saintpaul wild-type and *ΔycfR::cat* mutant was less notable (*P* = 0.035, see Table 2).

**Table 2:** Statistical analysis of *S. enterica* crystal violet attachment assays on glass and polystyrene surfaces.

Strain	ST LT2 WT^a^		SP WT^b^	
	Glass	Polystyrene	Glass	Polystyrene
**mutants**				
*S.* Typhimurium LT2 *ΔycfR::cat*	0.0063^c^	0.021		
*S.* Typhimurium LT2 *ΔyigG::cat*	NS	0.0003		
*S.* Typhimurium LT2 *ΔsirA::cat*	0.0007	0.009		
**complements**				
*S.* Typhimurium LT2 *ΔycfR::cat* (pJS-8)	0.023	NS		
*S.* Typhimurium LT2 *ΔyigG::cat* (pJS-16)	NS	0.006		
*S.* Typhimurium LT2 *ΔsirA::cat* (pJS-18)	NS	0.040		
**mutants**				
*S.* Saintpaul *ΔycfR::cat*			0.035	NS
*S.* Saintpaul *ΔyigG::cat*			NS	0.020
*S.* Saintpaul *ΔsirA::cat*			0.0025	<0.0001
**complements**				
*S.* Saintpaul *ΔycfR::cat* (pJS-10)			NS	NS
*S.* Saintpaul *ΔyigG::cat* (pJS-16)			NS	NS
*S.* Saintpaul *ΔsirA::cat* (pJS-18)			NS	0.010

a ST LT2 wild type strain under the same condition is used as a reference in *t*-test analysis.

b SP wild type strain under the same condition is used as a reference in *t-*test analysis.

c All values are given as probability (*P*). *P*>0.05 indicates not significant (NS).

In contrast, *ΔsirA::cat* in *S.* Saintpaul displayed significantly decreased attachment on both glass (Fig. 4A) and polystyrene surfaces (Fig. 4B) (*P* = 0.0025 and *P*<0.0001, respectively, see Table 2) whereas the complementation mutant restored its attachment phenotype similar to that of the wild-type, indicating that *sirA* is necessary for biofilm production in *S.* Saintpaul. Interestingly, deletion of *sirA* in *S.* Typhimurium caused a less considerable but still significant reduction in attachment to both surfaces; in-trans complementation of *sirA* did not restore the attachment phenotype as that of the wild-type *S.* Typhimurium. In addition, *ΔyigG::cat* in *S.* Typhimurium showed a significant reduction in attachment in polystyrene plates (*P* = 0.0003) but not in glass test tubes. A slight difference was also detected for the *ΔyigG::cat* in *S.* Saintpaul on polystyrene surface (significant at *P* = 0.02).

### Bacterial Attachment to Plant Surfaces

**Figure 5:**
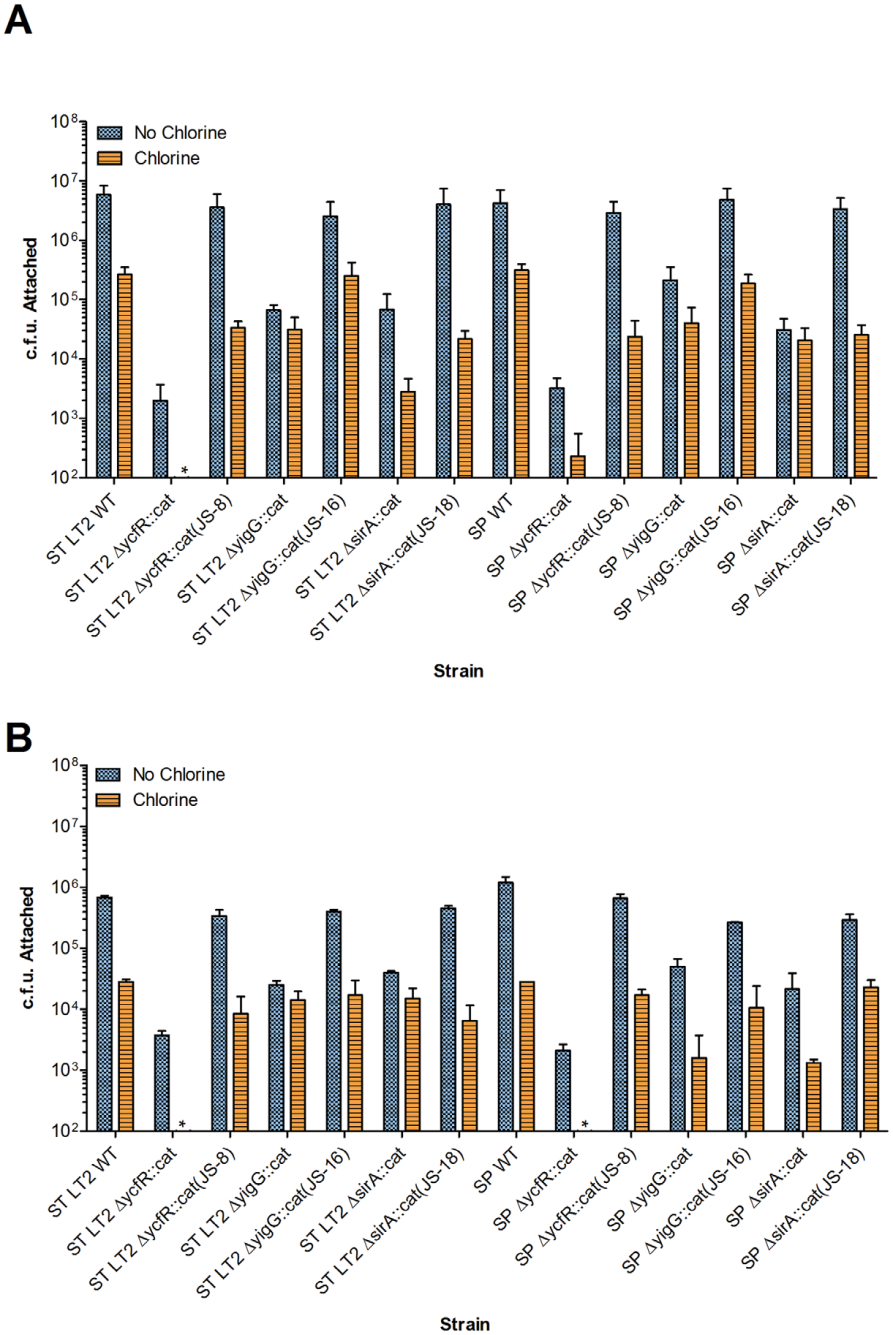
*S. enterica* attachment to fresh produce surface. A) Bacterial attachment to intact spinach leaves. B) Bacterial attachment to grape tomatoes. Attached bacteria were enumerated on XLD agar. Standard deviations represent three or four independent experiments. * indicates data is pΔresent but below the threshold for the graph. Detailed data analysis is given in Table 3.

In our recent studies, we showed that deletion of *ycfR* in *E. coli* O157:H7 decreased the attachment efficiency of the bacterium to baby spinach leaves under oxidative chlorine stress [18]. To determine if *ycfR* is involved in attachment of *S.* Typhimurium and *S.* Saintpaul to plant surfaces, we conducted attachment assays using both intact spinach leaves (Fig. 5A) and grape tomatoes (Fig. 5B). Deletion of *ycfR* in both serovars led to a decrease of 10^2^ to 10^3^ c.f.u. in attachment to spinach leaves and grape tomatoes after chlorine treatment. Interestingly, the *ΔycfR::cat* in *S.* Typhimurium was completely unable to attach to spinach leaves and tomatoes after chlorine treatment (*P*<0.0001, see Table 3). In addition, the attachment of *ΔycfR::cat* in *S.* Saintpaul to spinach leaves decreased more than 10^3^ c.f.u. after chlorine treatment (significant at *P*<0.0001). This mutant did not attach at all to tomatoes under the same stress conditions (significant at *P*<0.0001). In most cases, the complementation strains were able to restore the wild-type level of attachment after chlorine treatment. In the cases where the complement was not able to restore the wild-type attachment, we speculated that the plasmid used for complementation might interfere with how *S. enterica* responds to chlorine treatment, although this was not further tested in the current study. All the above collectively suggest that *ycfR* plays a critical role in bacterial plant surface attachment and subsequent chlorine resistance in both *S.* Typhimurium and Saintpaul.

**Table 3:** Statistical analysis of *S. enterica* spinach and tomato attachment assays with and without chlorine treatment.

Strain	ST LT2 WT^a^				SP WT^b^			
	Spinach		Tomato		Spinach		Tomato	
	−c	+^d^	−	+	−	+	−	+
**mutants**								
*S.* Typhimurium LT2 *ΔycfR::cat*	<0.0001^e^	<0.0001	<0.0001					
*S.* Typhimurium LT2*ΔyigG::cat*	<0.0001	<0.0001	<0.0001	0.025				
*S.* Typhimurium LT2 *ΔsirA::cat*	<0.0001	<0.0001	<0.0001	0.027				
**complements**								
*S.* Typhimurium LT2*ΔycfR::cat* (pJS-8)	NS	<0.0001	0.040	0.040				
*S.* Typhimurium LT2*ΔyigG::cat* (pJS-16)	0.010	NS	0.021	NS				
*S.* Typhimurium LT2*ΔsirA::cat* (pJS-18)	NS	<0.0001	0.044	0.030				
**mutants**								
*S.* Saintpaul *ΔycfR::cat*					<0.0001	<0.0001	0.003	<0.0001
*S.* Saintpaul *ΔyigG::cat*					0.0005	<0.0001	0.003	<0.0001
*S.* Saintpaul *ΔsirA::cat*					<0.0001	<0.0001	0.002	<0.0001
**complements**								
*S.* Saintpaul *ΔycfR::cat* (pJS-10)					NS	<0.0001	NS	0.044
*S.* SainΔtpaul *ΔyigG::cat* (pJS-16)					NS	NS	NS	NS
*S.* Saintpaul *ΔsirA::cat* (pJS-18)					NS	<0.0001	NS	NS

a ST LT2 wild type strain under the same condition is used as a reference in *t*-test analysis.

b SP wild type strain under the same condition is used as a reference in *t*-test analysis.

c (−) and ^d^(+) represent without and with chlorine treatment, respectively.

e All values are given as probability (*P*). *P*>0.05 indicates not significant (NS).

Attachment assays to intact spinach leaves (Fig. 5A) and grape tomatoes (Fig. 5B) were also performed with *ΔsirA::cat* and *ΔyigG::cat* mutants in *S.* Typhimurium and Saintpaul. Compared with their respective wild-type strains, *sirA* and *yigG* deletion mutants in both serovars showed decreased attachment to spinach leaves and grape tomatoes without chlorine treatment. The most noticeable reduction in the attachment to the plant surfaces after chlorine treatment was observed in *ΔsirA::cat* in *S.* Typhimurium and Saintpaul (significant at *P*<0.0001, see Table 3).

## Discussion

*S.* Typhimurium is a frequent contaminant of leafy vegetables and other fresh produce items [1], [6], [31]. The mechanisms that enable the survival of this pathogen during post-harvest processing, in particular with chlorinated water washes, have not been completely understood. In our studies of *E. coli* O157:H7, we demonstrated that *ycfR* was involved in bacterial chlorine resistance and survival on the surface of spinach leaves [18]. The results of this current study provided additional evidence to show that *ycfR*, which is the most up-regulated gene in *S. enterica* under chlorine stress [20], is also important for the chlorine resistance and attachment of *S. enterica* on plant surfaces.

*ycfR* encodes a putative membrane protein in *E. coli* K-12 and regulates biofilm production via a process that involves changing surface properties of the bacterial cell [17]. This gene also helps *E. coli* cope with multiple environmental stresses such as fluctuating temperature and pH [18], [20], [21]. In *E. coli* O157:H7, *ycfR* plays a critical role in the bacterial stress response and was most up-regulated under oxidative stress [20], [32]. Unlike in K-12, *ycfR* in O157:H7 does not alter the cell surface properties or biofilm formation [18]. Similar to *E. coli*, some functional differences of *ycfR* were observed between *S. enterica* serovars Typhimurium and Saintpaul. For instance, deletion of *ycfR* reduced attachment of *S.* Saintpaul to grape tomato to a greater extent than *S.* Typhimurium. *S.* Saintpaul has been predominantly associated with foodborne outbreaks linked to produce items such as tomatoes, jalapeño peppers, and alfalfa sprouts [9] but not leafy greens. It remains to be determined whether *ycfR* also plays a role in the attachment of other *S. enterica* serovars to various fruit and vegetable surfaces.

In our previous study on the global gene expression of *S.* Typhimurium under chlorine stress, *sirA* and *yigG* were up- and down-regulated, respectively [20]. *sirA* encodes Invasol SirA, a Type VI secretion protein of the EvpB family [16], [33]. This family of proteins consists of putative cytoplasmic, periplasmic, and outer membrane localizing proteins that are commonly found in Gram-negative organisms which are associated with eukaryotic cells in either a pathogenic or symbiotic manner [32]–[34]. The precise role and mode of action of this secretion system has not been thoroughly studied. Here we showed that *sirA* is involved in biofilm formation of *S. enterica* on both glass and polystyrene. Although the crystal violet staining method used here more readily quantifies attachment of bacteria and not necessarily biofilm formation, this technique was used as a generalized experiment to estimate bacterial biofilm productivity under different experimental conditions. In addition, we showed that both *sirA* and *yigG*, which encodes a putative inner membrane protein [35], are also involved in *S. enterica* attachment to spinach leaves and grape tomatoes. Admittedly, it remained unknown whether *ycfR*, *sirA* and *yigG* are involved in a single or multiple regulatory circuits in *S. enterica* or other pathogenic bacteria that control the oxidative stress response and plant surface attachment. More in-depth investigations may shed new light on the specific roles of these genes in the interaction of *S. enterica* with plant surfaces.

It should be noted that the findings from this study were based on laboratory-scale experiments of two *S. enterica* strains on baby spinach leaves and grape tomatoes. Future studies should include analysis of strains representing other *S. enterica* serotypes and strains, additional fresh produce items, and ideally scaled-up experimental approaches using actual processing lines to ascertain the roles of specific genes on *S. enterica* surface attachment in order to develop more effective and targeted strategies to minimize the contamination of this pathogen in fruit and vegetables, as well as on food contact surfaces during post-harvest minimal processing of fresh produce. We based this study on chlorine which is the most commonly used sanitizer in the current fresh produce industry. Use of other sanitizing agents or combined methods has been recommended to improve the killing of bacterial pathogens on fresh produce and minimize environmental and health hazards. Functional roles of *S. enterica* genes in response to these and other preventive control measures await to be further investigated in the future.

## Materials and Methods

### Bacterial Strains and Culture Conditions

Bacterial strains and mutants used in this study are listed in Table 1. *S.* Typhimurium strain LT2 (ATCC 19585) was obtained from the American Type Culture Collection (ATCC, Manassas, VA) and *S.* Saintpaul strain 99A3746 was kindly provided by the California State Department of Health. *E. coli* strains BW25113 (pKD46) and BW25141 (pKD3) were kindly provided by the *E. coli* Genetic Stock Center at Yale University. Vector pACYC177 was purchased from New England BioLabs, Beverly, MA. All mutants were derived from laboratory stocks in this study. Bacteria were grown in brain heart infusion (BHI) (Becton, Dickinson and Co., Franklin Lakes, NJ) supplemented with chloramphenicol (30 µg ml^−1^), ampicillin (50 µg ml^−1^), or kanamycin (30 µg ml^−1^) when necessary.

### Construction of in-frame Deletion and Complementation Mutants

In-frame gene deletions of *ycfR, sirA,* and *yigG* in *S.* Typhimurium and Saintpaul were generated using the lambda red recombinase method as described by Datsenko and Wanner [36]. The primer pairs used here are listed in Table S1. Briefly, 60-bp upstream and downstream regions of each target gene were fused to a chloramphenicol cartridge (*cat*) from pKD3 and transformed into competent *S. enterica* cells containing lambda recombinase plasmid pKD46 induced by 10 mM L-arabinose. Colonies of transformants with a deletion marked with the *cat* cassette were selected for on BHI agar plates containing chloramphenicol and verified by colony PCR and subsequent DNA sequencing.

Complementation mutants were constructed by cloning a fragment containing the original gene along with its promoter-region into the vector pACYC177. The *Bam*HI restriction site was used to obtain complementation vectors pJS-8, pJS-16, and pJS-18, which harbored gene *ycfR*, *yigG* and *sirA*, respectively. The vector was subsequently transformed into competent *S. enterica* deletion mutants. Colonies were selected on BHI agar plates containing kanamycin and verified by colony PCR and DNA sequencing.

### Measurement of Total and Free Chorine in BHI Broth

The total and free chlorine concentrations were measured at room temperature using ChloroSense (Palintest Limited, Tyne & Wear, England) according to the manufacturer’s instructions. Seventy ml BHI broth was supplemented with 2.7, or 3.0 µl per ml of a 13% fresh stock sodium hypochlorite solution, resulting in final free chlorine concentrations of 0.05±0.02, and 0.14±0.04 ppm in the broth, respectively.

### Bacterial Growth Under Chlorine Stress

Growth curves of *S. enterica* at 37°C were performed using a Bioscreen C automatic growth curve system (Growth Curves, Piscataway, NJ). Overnight cultures of *S. enterica* in BHI broth were normalized to an OD_600_ of 0.8. A 1∶10,000 dilution was aliquoted into BHI broth supplemented with 0.05±0.02 and 0.14±0.04 ppm free chlorine. A 200-µl aliquot was loaded in triplicate into a 100-well honeycomb plate for analysis. Uninoculated BHI broth was used as a negative control. Bacterial growth was monitored by recording the cell turbidity every 5 min, after a 10 s shaking period, over a period of 48 h. All experiments were repeated at least three times using independent cultures for statistical analysis.

### Aggregation Assay

Aggregation assays were conducted based on the method by Yonezawa *et al.* [37]. Briefly, bacteria were grown overnight at 37°C with shaking, centrifuged at 4,000 rpm for 20 min, washed with 5 ml PBS, and finally resuspended in 5 ml PBS. The bacteria were then incubated at room temperature without shaking and the OD_600_ was measured and recorded at 1, 2, 3, 4, and 24 h, respectively. The percent aggregation was calculated as (OD_600_ before incubation - OD_600_ after incubation)/OD_600_ before incubation × 100. Experiments were repeated at least three times with triplicate samples for statistical analysis.

### Cell Surface Hydrophobicity Assay

Cell adherence to hexane was measured as previously described by Deng *et al*. [18]. Briefly, overnight cultures incubated at 37°C were centrifuged and resuspended in PBS containing differing amounts of hexane. After incubation at room temperature for 1 h, the hexane phase was removed and OD_600_ of the remaining aqueous suspension was measured. Hydrophobicity is represented as the calculated percentage of bacteria remaining in the aqueous phase. Experiments were repeated at least three times with triplicate samples for statistical analysis.

### Crystal Violet Attachment Assay

Crystal violet attachment assay was performed as previously described by Deng *et al*. [18]. Briefly, individual bacterial cultures were grown overnight at 37°C in BHI broth, normalized, and then grown overnight without shaking in glass test tubes or polystyrene 96-well microtiter plates. After overnight growth, the liquid was removed, and attached bacteria remaining in the test tubes or wells were washed three times with PBS and incubated with 3 ml (for test tubes) or 200 µl (for microtiter plates) of 1% crystal violet for 15 min. The test tubes and plates were then washed three times with PBS and incubated with 95% ethanol for 20 min. The OD_570_ readings, which reflect the amount of attached bacteria, were measured. All experiments were performed at least three times with triplicate samples for statistical analysis.

### Bacterial Attachment to Spinach Leaf and Grape Tomato

Attachment assays were conducted for each wild-type strain and its corresponding deletion and complementation mutants, based on the method by Deng *et al*. [18] with minor modifications. Baby spinach and grape tomatoes were purchased from a local retail grocer. *S. enterica* cultures were grown overnight in BHI at 37°C with shaking. All cultures were normalized to 1×10^8^ c.f.u./ml and 1 ml was added into a 50-ml conical tube containing 45 ml PBS. For each experiment, six pieces of intact spinach leaves (approximately 1 g each) were placed into the conical tubes and incubated with *S. enterica* culture for 10 min. After incubation, leaves were pulled out and air dried in a biohazard cabinet in sterile petri dishes for 1 h. Three leaves was washed three times with PBS as the untreated control; the other three leaves was immersed in a 50 ppm aqueous chlorine solution (made from a 13% sodium hypochlorite stock solution) for 10 s and the reaction was stopped by adding 4.5 ml of 1 M sodium thiosulfate. The chlorine-treated leaves were then washed three times with PBS. To recover attached bacteria, five sterile 6-mm glass beads were added to the leaves in 50-ml conical tubes containing 10 ml PBS and vortexed vigorously for 1 min. Serial dilutions of the eluted bacteria were plated on XLD agar (Becton Dicksinson and Co.) in duplicate. The plates were incubated at 37°C for 24 h before c.f.u. were enumerated. Attachment assays with tomatoes were performed similarly using six intact grape tomatoes (approximately 8 g each) for each experiment. All attachment experiments were conducted independently for at least three times.

### Phylogenetic Analysis

Gene sequences of *ycfR*, *sirA*, and *yigG* in *S. enterica* and their homologs in other bacterial genomes were retrieved from GenBank under accession numbers as shown in Fig. 1. Multiple gene alignments were performed using MUSCLE 3.7 and Gblocks 0.91 b modules of Phylogeny.fr. Phylogenetic analysis was performed using PhyML 3.0 aLRT and cladograms were generated using TreeDyn 198.3 module of Phylogeny.fr. All the above software and modules are freely available at http://www.phylogeny.fr/ [23].

### Statistical Analysis

Student’s *t*-test analysis was performed using GraphPad Prism software package (GraphPad Software, Version 5).

## Supporting Information

Figure S1 **Gene sequence alignment of** ***ycfR*** **,** ***sirA*** **, and** ***yigG*** **in** ***S. enterica*** **and their homologs in related bacterial species.** Multiple gene sequence alignments of *ycfR* (A), *sirA* (B), and *yigG* (C).
(DOCX)

Table S1 **Primers used in this study.** A list of all primers used in the study along with sequences.
(DOCX)
