# Supplementary material for: Genes ycfR, sirA and yigG Contribute to the Surface Attachment of Salmonella enterica Typhimurium and Saintpaul to Fresh Produce
Source: PLoS One. 2013 Feb 22;8(2):e57272. doi: 10.1371/journal.pone.0057272 (PMC3579871; doi:10.1371/journal.pone.0057272)
Supplement: Figure S1 — Gene sequence alignment of ycfR , sirA , and yigG in S. enterica and their homologs in related bacterial species. Multiple gene sequence alignments of ycfR (A), sirA (B), and yigG (C). (DOCX) [file pone.0057272.s001.docx]

Supplemental Figure

Figure S1A

gi|3775200 ------------------------------------------------------------

gi|3876054 ------------------------------------------------------------

gi|4299568 ------------------------------------------------------------

gi|4278076 ------------------------------------------------------------

gi|4237004 ------------------------------------------------------------

gi|4278030 ------------------------------------------------------------

gi|3876104 ------------------------------------------------------------

gi|1582925 ------------------------------------------------------------

gi|4917599 ------------------------------------------------------------

gi|2388994 ------------------------------------------------------------

gi|3878254 ------------------------------------------------------------

gi|3399980 ------------------------------------------------------------

gi|1676339 ------------------------------------------------------------

gb|ABAM020 ------------------------------------------------------------

gi|3865892 atgattaatttatcgatcgttaagttaatgcttgcgacacccaTtCaTcTgctaaAggtC

gi|1944422 ------------------------------------------------------------

gi|4017615 ------------------------------------------------------------

gi|3976551 ------------------------------------------------------------

gi|2889328 ------------------------------------------TTACTTgTAGATaACaGC

gi|4227797 ------------------------------------------TTAtTTATAaATTACtGC

gi|4074667 ------------------------------------------TTAtTTATAaATTACtGC

gi|8277538 ------------------------------------------TTAtTTATAaATTACtGC

gi|8254261 ------------------------------------------TTAtTTATAaATTACtGC

gi|1615019 ------------------------------------------TTACTTgTAGATTACCGC

gi|3789581 ------------------------------------------TTACTTATAGATTACCGC

gi|2078555 ------------------------------------------TTACTTATAGATTACCGC

gi|1972473 ------------------------------------------TTACTTATAGATTACCGC

gi|1973612 ------------------------------------------TTACTTATAGATTACCGC

gi|3775200 ------ATGAAAAACGTAAAAACCCTCATCGCTGCGGCGAT---------TTTAAGCTCC

gi|3876054 ------ATGAAAAACGTAAAAACCCTCATCGCTGCGGCGAT---------TTTAAGCTCa

gi|4299568 ------ATGAAAAACGTAAAAACCCTCATCGCTGCGGCGAT---------TTTAAGCTCC

gi|4278076 ------ATGAAAAACGTAAAAACCCTCATCGCTGCGGCGAT---------TTTAAGCTCC

gi|4237004 ------ATGAAAAACGTAAAAACCCTCATCGCTGCGGCGAT---------TTTAAGCTCC

gi|4278030 ------ATGAAAAACGTAAAAACCCTCATCGCTGCGGCGAT---------TTTAAGCTCC

gi|3876104 ------ATGAAAAACGTAAAAACCCTCATCGCTGCGGCGAT---------TTTAAGCTCC

gi|1582925 ------ATGAAAAACGTAAAAACCCTCATCGCTGCGGCGAT---------TTTAAGCTCC

gi|4917599 ------ATGAAAAACGTAAAAACCCTCATCGCTGCGGCGAT---------TTTAAGCTCC

gi|2388994 ------ATGAAAAACGTAAAAACCCTCATCGCTGCGGCGAT---------TTTAAGCTCC

gi|3878254 ------ATGAAAAACGTAAAAACCCTCATCGCTGCGGCGAT---------TTTAAGCTCC

gi|3399980 ------ATGAAAAACGTAAAAACCCTCATCGCTGCcGCagT---------TcTtgGtTCg

gi|1676339 ------ATGAAAAACGTAAAAACCCTCATCGCTGCtGCcgT---------acTgAGtTCg

gb|ABAM020 ------ATGAAAAACGTAAAAACCCTCATCGCTGCtGCcgT---------acTgAGtTCg

gi|3865892 atcACtATGAAAAACGTAAAAACCCTCATCGCTGCtGCGgT---------acTgAGtTCg

gi|1944422 ------ATGAAAAACGTAAAAACCCTCATCGCTGCtGCGgT---------acTgAGtTCg

gi|4017615 ------ATGAAAAACGTAAAAACCCTCATCGCTGCaGCcgT---------acTgAGtTCa

gi|3976551 ------ATGAAAAACGTtAAAACaCTCgctatcGCcGCtgT---------TcTtAGCTCa

gi|2889328 gGTgCCgTGAAgggtaTtcggACCg--gTtaCcGaaGtaATACGGAAtGAggTggcgcCC

gi|4227797 tGTtCCATGgAgggtaTtcggACCg--gTtaCaGaaGtaATACGGAAAGATTTtgcgcCC

gi|4074667 tGTtCCATGgAgggtaTtcggACCg--gTtaCaGaaGtaATACGGAAAGATTTtgcgcCC

gi|8277538 tGTtCCATGgAgggtaTtcggACCg--gTtaCaGaaGtaATACGGAAAGATTTtgcgcCC

gi|8254261 tGTtCCATGgAgggtaTtcggACCg--gTtaCaGaaGtaATACGGAAAGATTTtgcacCC

gi|1615019 CGTACCgTGAAgggtaTtcgggCCa--gTgataGaGGtaATACGGAAAGAcTTcgcgcCC

gi|3789581 CGTACCgTGgAgggtaTtcgggCCg--gTaaCaGaGGtaATACGGAAAGAcTTcgcCcCC

gi|2078555 CGTACCgTGAAgggtaTtcgggCCg--gTaaCaGaGGtaATACGGAAAGAcTTcgcCcCC

gi|1972473 CGTACCgTGgAgggtaTtcgggCCg--gTaaCaGaGGtaATACGGAAAGAcTTcgcCcCC

gi|1973612 CGTACCgTGAAgggtaTtcgggCCg--gTaaCaGaGGtaATACGGAAAGAcTTcgcCcCC

gi|3775200 ATgTCATTtGCCAaCTTTGCGGCTGTcGAAGTTCAGtCAACGCCAGA---------AGGc

gi|3876054 ATgTCATTtGCCAGCTTTGCGGCTGTcGAAGTTCAGtCAACGCCAGA---------AGGc

gi|4299568 ATgTCATTtGCCAGCTTTGCGGCTGTcGAAGTTCAGtCAACGCCAGA---------AGGc

gi|4278076 ATgTCATTtGCCAGCTTTGCGGCTGTcGAAGTTCAGtCAACGCCAGA---------AGGc

gi|4237004 ATgTCATTtGCCAGCTTTGCGGCTGTcGAAGTTCAGtCAACGCCAGA---------AGGc

gi|4278030 ATgTCATTtGCCAGCTTTGCGGCTGTcGAAGTTCAGtCAACGCCAGA---------AGGc

gi|3876104 ATgTCATTtGCCAGCTTTGCGGCTGTcGAAGTTCAGtCAACGCCAGA---------AGGc

gi|1582925 ATgTCATTtGCCAGCTTTGCGGCTGTcGAAGTTCAGtCAACGCCAGA---------AGGc

gi|4917599 ATgTCATTtGCCAGCTTTGCGGCTGTcGAAGTTCAGtCAACGCCAGA---------AGGc

gi|2388994 ATgTCATTtGCCAGCTTTGCGGCTGTcGAAGTTCAGtCAACGCCAGA---------AGGc

gi|3878254 ATgTCATTtGCCAGCTTTGCGGCgGTcGAAGTTCAGtCAACGCCAGA---------AGGc

gi|3399980 cTCTCgTTCGCCAGtTTTGCtGCcGTTGAAGTgCAGGCAACGCCAGA---------AGGT

gi|1676339 cTCTCATTCGCCAGCTTcGCcGCcGTTGAAGTTCAGGCAACGCCAGA---------AGGT

gb|ABAM020 cTCTCATTCGCCAGCTTcGCcGCcGTTGAAGTTCAGGCAACGCCAGA---------AGGT

gi|3865892 cTCTCATTCGCCAGCTTcGCcGCcGTTGAAGTTCAGGCAACGCCAGA---------AGGT

gi|1944422 cTCTCATTCGCCAGCTTcGCcGCcGTTGAAGTTCAGGCAACGCCAGA---------AGGT

gi|4017615 cTtTCtTTCGCaAGCgTTGCtGCTGTacAAGTTCAGtCAACGCCAGc---------cGaT

gi|3976551 cTCTCtTTCGCCAGCTTcGCcGCTGTaGAAGTTCAGtCAACGCCgGc---------gGGT

gi|2889328 ATCTCATcCGCt--tTcTGCGcCaGcTGAtcTTCcaGCgAgcCCAGgTTaGTCCCGgcag

gi|4227797 ATCTCATcCGCt--tTaTGCGcCaGcTGctcTTCcaGCgAtcCCAGATTtGTCCCcgcGT

gi|4074667 ATCTCATcCGCt--tTTTGCGcCaGcTGctcTTCcaGCgAtcCCAGATTtGTCCCcgcGT

gi|8277538 ATCTCATcCGCt--tTTTGCGcCaGcTGctcTTCcaGCgAtcCCAGATTtGTCCCcgcGT

gi|8254261 ATCTCATcCGCt--tTTTGCGcCaGcTGctcTTCcaGCgAtcCCAGATTtGTCCCcgctT

gi|1615019 ATCTCtTcCGCt--tTcTGCGctaGcTGAtcTTCcaGCgAtcCCAGATTCGTgCCGccaT

gi|3789581 ATtTCcTgCGCt--tTTTGCGcCaGcTGAtcTTCcaGCgAgcCCAGATTCGTgCCGccaT

gi|2078555 ATCTCcTgCGCt--tTTTGCGcCaGcTGAtcTTCcaGCgAgcCCAGATTCGTgCCGccaT

gi|1972473 ATCTCcTgCGCt--tTTTGCGcCaGcTGAtcTTCcaGCgAgcCCAGATTCGTgCCGccaT

gi|1973612 ATCTCcTgCGCt--tTTTGCGcCaGcTGAtcTTCcaGCgAgcCCAGATTCGTgCCGccaT

gi|3775200 CAACAAAAAgTCGGtACtATcagTGCTAACGCGGGGACAAATCTGGGATCGCTGGAAGAg

gi|3876054 CAACAAAAAgTCGGtACtATcagTGCTAACGCGGGGACAAATCTGGGATCGCTGGAAGAg

gi|4299568 CAACAAAAAgTCGGtACAATcagTGCTAACGCGGGGACAAATCTGGGATCGCTGGAAGAg

gi|4278076 CAACAAAAAgTCGGtACAATcagTGCTAACGCGGGGACAAATCTGGGATCGCTGGAAGAg

gi|4237004 CAACAAAAAgTCGGtACAATcagTGCTAACGCGGGGACAAATCTGGGATCGCTGGAAGAg

gi|4278030 CAACAAAAAgTCGGtACAATcagTGCTAACGCGGGGACAAATCTGGGATCGCTGGAAGAg

gi|3876104 CAACAAAAAgTCGGtACAATcagTGCTAACGCGGGGACAAATCTGGGATCGCTGGAAGAg

gi|1582925 CAACAAAAAgTCGGtACAATcagTGCTAACGCGGGGACAAATCTGGGATCGCTGGAAGAg

gi|4917599 CAACAAAAAgTCGGtACAATcagTGCTAACGCGGGGACAAATCTGGGATCGCTGGAAGAg

gi|2388994 CAACAAAAAgTCGGtACAATcagTGCTAACGCGGGGACAAATCTGGGATCGCTGGAAGAg

gi|3878254 CAACAAAAAgTCGGtACAATcagTGCTAACGCGGGGACAAATCTGGGATCGCTGGAAGAg

gi|3399980 CAACAgAAATTCGGtACgATTTCTGCgAAtGgcGGaACtAATCTcGGATCGCTGGAAGAT

gi|1676339 CAACAgAAgTTCGGCACcATTTCcGCgAAtGgcGGcACgAATCTGGGcTCGCTGGAAGAT

gb|ABAM020 CAACAgAAgTTCGGCACcATTTCcGCgAAtGgcGGcACgAATCTGGGcTCGCTGGAAGAT

gi|3865892 CAACAgAAgTTCGGCACcATTTCcGCgAAtGgcGGcACgAATCTGGGcTCGCTGGAAGAT

gi|1944422 CAACAgAAgTTCGGCACcATTTCcGCgAAtGgcGGcACgAATCTGGGcTCGCTGGAAGAT

gi|4017615 CAgCAtAAAgTCGGgACgATcTCTGCgtctGCcGGGACtAAcCTGGGtTCaCTGGAAGAT

gi|3976551 CAgCAtAAAaTCGGtACcgTTTCcGCctcaGCcGGGACcAAcCTGGGtTCGCTGGAAGAT

gi|2889328 aAgCAgAgATggtGCctAcTTTaTGCTgACcCG---------CTGGGgTgGactGcAccT

gi|4227797 tAgCActgATagtaCcgAcTTTtTGtTggCct---------TCTGGcgTtGactGAActT

gi|4074667 tAgCActgATTgtaCcgAcTTTtTGtTggCct---------TCTGGcgTtGactGAActT

gi|8277538 tAgCActgATTgtaCcgAcTTTtTGtTggCct---------TCTGGcgTtGactGAActT

gi|8254261 tAgCActgATTgtaCcgAcTTTtTGtTggCct---------TCTGGcgTtGactGAActT

gi|1615019 tcgCggAAATTgtaCcgAAcTTCTGtTggCct---------TCcGGcgTtGCctGgActT

gi|3789581 tcgCggAAATggtGCcgAAcTTCTGtTgACct---------TCTGGcgTtGCctGAActT

gi|2078555 tcgCggAAATggtaCcgAAcTTCTGtTgACct---------TCTGGcgTtGCctGAActT

gi|1972473 tcgCggAAATggtGCcgAAcTTCTGtTgACct---------TCTGGcgTtGCctGAActT

gi|1973612 tcgCggAAATggtGCcgAAcTTCTGtTgACct---------TCTGGcgTtGCctGAActT

gi|3775200 CAGCTGGCGCAAAA--AGCGGATGAGATGGGCGCAAAATCTTTCCGTATTACTTCTGTAA

gi|3876054 CAGCTGGCGCAAAA--AGCGGATGAGATGGGCGCAAAATCTTTCCGTATTACTTCTGTAA

gi|4299568 CAGCTGGCGCAAAA--AGCGGATGAGATGGGCGCAAAATCTTTCCGTATTACTTCTGTAA

gi|4278076 CAGCTGGCGCAAAA--AGCGGATGAGATGGGCGCAAAATCTTTCCGTATTACTTCTGTAA

gi|4237004 CAGCTGGCGCAAAA--AGCGGATGAGATGGGCGCAAAATCTTTCCGTATTACTTCTGTAA

gi|4278030 CAGCTGGCGCAAAA--AGCGGATGAGATGGGCGCAAAATCTTTCCGTATTACTTCTGTAA

gi|3876104 CAGCTGGCGCAAAA--AGCGGATGAGATGGGCGCAAAATCTTTCCGTATTACTTCTGTAA

gi|1582925 CAGCTGGCGCAAAA--AGCGGATGAGATGGGCGCAAAATCTTTCCGTATTACTTCTGTAA

gi|4917599 CAGCTGGCGCAAAA--AGCGGATGAGATGGGCGCAAAATCTTTCCGTATTACTTCTGTAA

gi|2388994 CAGCTGGCGCAAAA--AGCGGATGAGATGGGCGCAAAATCTTTCCGTATTACTTCTGTAA

gi|3878254 CAGCTGGCGCAAAA--AGCGGATGAGATGGGCGCAAAATCTTTCCGTATTACTTCTGTAA

gi|3399980 CAaCTGGCaCAgAA--AGCGGAaGAaATGGGCGCgAAgTCTTTCCGTATTACcTCTGTtA

gi|1676339 CAGCTGGCGCAAAA--AGCGcAgGAGATGGGgGCgAAgTCTTTCCGTATTACcTCTGTtA

gb|ABAM020 CAGCTGGCGCAAAA--AGCGcAgGAGATGGGgGCgAAgTCTTTCCGTATTACcTCTGTtA

gi|3865892 CAGCTGGCGCAAAA--AGCGcAgGAGATGGGgGCgAAgTCTTTCCGTATTACcTCTGTtA

gi|1944422 CAGCTGGCGCAAAA--AGCGcAgGAGATGGGgGCgAAgTCTTTCCGTATTACcTCTGTtA

gi|4017615 CAGCTGGCaCAAAA--AGCGGAaGcaATGGGtGCAAAATCcTaCCGcATcACcTCTGTgA

gi|3976551 CAGCTGGCGCAAAA--AGCGGAaGAGATGGGCGCAAccTCTTaCCGTATTACgTCcGTAA

gi|2889328 CAaCaGcgGCAAAgCTgGCGaAaGAGAgtGagctAAgA---------AcggCggCaaTAg

gi|4227797 CgaCaGcCGCAAAgCTgGCaaATGAcATtGagcttAAA---------ATcgCcgCaGcgA

gi|4074667 CgaCaGcCGCAAAgCTgGCaaATGAcATGGagcttAAA---------ATcgCcgCaGcgA

gi|8277538 CgaCaGcCGCAAAgCTgGCaaATGAcATGGagcttAAA---------ATcgCcgCaGcgA

gi|8254261 CgaCaGcCGCAAAgCTgGCaaATGAcATGGagcttAAA---------ATcgCcgCaGcgA

gi|1615019 CcaCgGcgGCgAAACTgGCGaATGAGAgcGaactAAA---------TAccACagCaGcgA

gi|3789581 CAaCgGcgGCgAAgCTgGCGaATGAGA-----GCgAA----cTCaGTAccgCagCaGcgA

gi|2078555 CAaCgGcgGCgAAgCTgGCGaATGAGA-----GCgAA----cTCaGTAcggCagCaGcgA

gi|1972473 CAaCgGcgGCgAAgCTgGCGaATGAGA-----GCgAA----cTCaGTAcggCagCaGcgA

gi|1973612 CAaCgGcgGCgAAgCTgGCGaATGAGA-----GCgAA----cTCaGTAccgCagCaGcgA

gi|3775200 CC--GGTCCGAATACCCTcCATGGAA-----------------------------CAGCA

gi|3876054 CC--GGTCCGAATACCCTcCATGGAA-----------------------------CAGCA

gi|4299568 CC--GGTCCGAATACCCTcCATGGAA-----------------------------CAGCA

gi|4278076 CC--GGTCCGAATACCCTcCATGGAA-----------------------------CAGCA

gi|4237004 CC--GGTCCGAATACCCTcCATGGAA-----------------------------CAGCA

gi|4278030 CC--GGTCCGAATACCCTcCATGGAA-----------------------------CAGCA

gi|3876104 CC--GGTCCGAATACCCTcCATGGAA-----------------------------CAGCA

gi|1582925 CC--GGTCCGAATACCCTcCATGGAA-----------------------------CAGCA

gi|4917599 CC--GGTCCGAATACCCTcCATGGAA-----------------------------CAGCA

gi|2388994 CC--GGTCCGAATACCCTcCATGGAA-----------------------------CAGCA

gi|3878254 CC--GGTCCGAATACCCTcCATGGAA-----------------------------CAGCA

gi|3399980 Cg--GGTCCaAAcACaCTTCAcGGtA-----------------------------CcGCg

gi|1676339 CC--GGcCCGAATACCCTcCAcGGtA-----------------------------CgGCg

gb|ABAM020 CC--GGcCCGAATACCCTcCAcGGtA-----------------------------CgGCg

gi|3865892 CC--GGcCCGAATACCCTTCAcGGtA-----------------------------CgGCg

gi|1944422 CC--GGcCCGAATACCCTTCAcGGtA-----------------------------CgGCg

gi|4017615 CC--GGTCCtAAcACgCTgCAcGGcA-----------------------------CgGCt

gi|3976551 CC--GGTCCtAAcACCCTTCAcGGgA-----------------------------CtGCg

gi|2889328 CCAGaGTattAAcggttTTCAT--------------------------------------

gi|4227797 tgAGGGTttttAcgtttTTCAT--------------------------------------

gi|4074667 tgAGGGTttttAcgtttTTCAT--------------------------------------

gi|8277538 tgAGGGTttttAcgtttTTCAT--------------------------------------

gi|8254261 tgAGGGTttttAcgtttTTCAT--------------------------------------

gi|1615019 tgAGGaTttttAcgtttTTCATaGtc----------------------------------

gi|3789581 tgAGGtTttttAcgtttTTCATaGtgatgacctttagcagatgaatgggtgtcgcaAGCA

gi|2078555 tgAGGtTttttAcgtttTTCAT--------------------------------------

gi|1972473 tgAGGGTttttAcgtttTTCAT--------------------------------------

gi|1973612 tgAGGGTttttAcgtttTTCAT--------------------------------------

gi|3775200 GTAATTT--------ATAAATAA-----

gi|3876054 GTAATTT--------ATAAATAA-----

gi|4299568 GTAATTT--------ATAAATAA-----

gi|4278076 GTAATTT--------ATAAATAA-----

gi|4237004 GTAATTT--------ATAAATAA-----

gi|4278030 GTAATTT--------ATAAATAA-----

gi|3876104 GTAATTT--------ATAAATAA-----

gi|1582925 GTAATTT--------ATAAATAA-----

gi|4917599 GTAATTT--------ATAAATAA-----

gi|2388994 GTAATTT--------ATAAATAA-----

gi|3878254 GTAATTT--------ATAAATAA-----

gi|3399980 GTtATcT--------AcAAgTAA-----

gi|1676339 GTAATcT--------ATAAgTAA-----

gb|ABAM020 GTAATcT--------ATAAgTAA-----

gi|3865892 GTAATcT--------ATAAgTAA-----

gi|1944422 GTAATcT--------ATAAgTAA-----

gi|4017615 GTcATcT--------AcAAATAA-----

gi|3976551 GTtATcT--------AcAAATAA-----

gi|2889328 ----------------------------

gi|4227797 ----------------------------

gi|4074667 ----------------------------

gi|8277538 ----------------------------

gi|8254261 ----------------------------

gi|1615019 ---------------AT-----------

gi|3789581 tTAAcTTaacgatcgATAAATtAatcat

gi|2078555 ----------------------------

gi|1972473 ----------------------------

gi|1973612 ----------------------------

*Legend:
Similar residues are colored as the most conserved one (according to BLOSUM62).
Average BLOSUM62 score: Max: 3.0 Mid: 1.5 Low: 0.5*

Figure S1B

gi|3789825 ------------ATGgcAaACagtaATAtgCAgGCAaCcgaCGCggTtgctCAggatacc

gi|3896866 atggccgaattgATGaaAgACccGCAggccCAgGCAGacgcCGCgaTCgAACA-------

gi|2956804 -----------------------------TCAgGCgGCTT---CcTTCAcgag-------

gi|4017615 -----------------------------TCAcGCgtCTTTCGtcTTCAgcgA-------

gi|3898390 -----------------------------TtAtGCAaCaT---gtTTtAAAgA-------

gi|1676339 ------------------------------------------------------------

gi|3796992 ------------------------------------------------------------

gb|ABAM020 ------------------------------------------------------------

gi|3927414 ------------------------------------------------------------

gi|3635575 ------------------------------------------------------------

gi|3536257 ------------------------------------------------------------

gi|3751174 ---------------------gtGCATAaaCAtGCgGCTTTCtatcTtgAACA-------

gi|3789825 gcctccgcAtCCGGtgaaTTtGatgCgtTgCTgAAtcAggCCtTCcGACCcaaGActacc

gi|3896866 --------AcCgaGCgaCTTCGCcgCCcTgCTCctgcAAgagtTCAaACCcaaGAcCgag

gi|2956804 -----------CGGCAGCTTCGCaACgAgACgCAAaGAAACCGTCAGACCTTCGAGC---

gi|4017615 -----------CGGCAGtTTaGaaACCAgACgCAggGAAACgGTCAGgCCTTCcAGC---

gi|3898390 -----------aGGCAGtTTCGCgACCATtCTtAAcGAcACCGTtAatCCTTCcAGC---

gi|1676339 ------------------------------------------------------------

gi|3796992 ------------------------------------------------------------

gb|ABAM020 ------------------------------------------------------------

gi|3927414 ------------------------------------------------------------

gi|3635575 ------------------------------------------------------------

gi|3536257 ------------------------------------------------------------

gi|3751174 ------------------------------------------------------------

gi|3789825 CAgGCggcaaAaGCcGTGGAAgcCGCgGTGCaGACGCtGGcgcAACaggcgCTGGcgAac

gi|3896866 CAcGCccgcgAgGCgGTGGAAAgCGCcGTGCGcACcCtGGccGAACaggcCCTGagcAag

gi|2956804 ----------------TGGAAA------TGCGGACGCAGGAAGAAtTTCGCCTGGTAATA

gi|4017615 ----------------TGGtAg------TGtGGACGCAGGAAGAACTTCGCggcGTAgTA

gi|3898390 ----------------TGaAAA------TGCGGgCGCAaaAAGAAtTTCGCCTGaTAATA

gi|1676339 ------------------------------------------------------------

gi|3796992 ------------------------------------------------------------

gb|ABAM020 ------------------------------------------------------------

gi|3927414 ------------------------------------------------------------

gi|3635575 ------------------------------------------------------------

gi|3536257 ------------------------------------------------------------

gi|3751174 ------------------------------------------------------------

gi|3789825 ACgAtcAccgTCagCgAtGaCgcCtAtAAaagCATCaGCgCtATtATtGCgcagATCGAC

gi|3896866 ACCgaGcTgaTCTcCAACGaCgcgatCAAatcCATCGaatCgATcATcGCcgcaATCGAC

gi|2956804 tCCcGGATTtcCcTCgACGTCcTCgACgAccACtTCGGCg--------------------

gi|4017615 ACCcGGgTTgTCTTCAAtcTCcTgcACctgcACtTCcGCA--------------------

gi|3898390 ACCAGGATTccCTTCcACaTCtTCcACAAtgACcTCGGCA--------------------

gi|1676339 ------------------------------------------------------------

gi|3796992 ------------------------------------------------------------

gb|ABAM020 ------------------------------------------------------------

gi|3927414 ------------------------------------------------------------

gi|3635575 ------------------------------------------------------------

gi|3536257 ------------------------------------------------------------

gi|3751174 ------------------------------------------------------------

gi|3789825 tttAAaCTgACCGaaCAGaTCAAaCTGATCcTGCAaCAtcCCGAcTggCAGaAgCTGGAA

gi|3896866 gccAAgCTcACCGcgCAGgTCAAcCTGATCaTGCAtCAcgCCGAtTtcCAGcAaCTGGAA

gi|2956804 ------------------------------------------------------------

gi|4017615 ------------------------------------------------------------

gi|3898390 ------------------------------------------------------------

gi|1676339 ------------------------------------------------------------

gi|3796992 ------------------------------------------------------------

gb|ABAM020 ------------------------------------------------------------

gi|3927414 ------------------------------------------------------------

gi|3635575 ------------------------------------------------------------

gi|3536257 ------------------------------------------------------------

gi|3751174 ------------------------------------------------------------

gi|3789825 tcCtCgtgGcGCGGTaTGgagcAtcTGGTTTaCAACacCGAGacCGACGaAaaGCtGaAa

gi|3896866 agCGCCtgGcGCGGcCTGCactACcTGGTcaaCAACacgGAaacCGACGagcaGCtGaAg

gi|2956804 ---GCgGCcAGCGGTCgaCGtGcCTTGGTTTCCtggGACGAaTTCGcgGGATCagCGTcg

gi|4017615 ---GCCGCcAGCGGTtTaCGgGACTTGGTTTCCtgaGAgGAGTTCGcCGGgTCaCCGTcc

gi|3898390 ---GCCGCGAGCGGgCgGCGaGACTTGGTTTCtAAtGACGAGTTaGtaGGATCGgCaTcg

gi|1676339 ------------------------------------------------------------

gi|3796992 ------------------------------------------------------------

gb|ABAM020 ------------------------------------------------------------

gi|3927414 ------------------------------------------------------------

gi|3635575 ------------------------------------------------------------

gi|3536257 ------------------------------------------------------------

gi|3751174 --------------------------GGaTTCtAAC---------------------TAt

gi|3789825 AttcgcTTCATGAAtCtGTCAaaagatgAatTGCgGCgcAaCaTgaaGCgtTacAAgGGC

gi|3896866 AtccgcgTgATGAgCatcgCcaaggGCgACCTGCacaAgAcCcTgaaGaagTTcAAgGGC

gi|2956804 ACgTAgTTCATGAcCCAtTCATTgAGCCACt-GCTGCATtTCgTCGCGCTCcTtgAAtGC

gi|4017615 ACgTAgTTCATtAcCCAGTCgTTCAGCCAgC-GtTcCATcTCtTCGCGCTCgcggAAGGa

gi|3898390 ACaTAaTTCATGAtCCAGTCATTCAGCCAgC-GCTGCATATCgTCGCGCTCTTtAAAGGt

gi|1676339 ------------------------------------------------------------

gi|3796992 ------------------------------------------------------------

gb|ABAM020 ------------------------------------------------------------

gi|3927414 ------------------------------------------------------------

gi|3635575 ------------------------------------------------------------

gi|3536257 ------------------------------------------------------------

gi|3751174 AttTAtgTtATGgAtC--------------------------------------------

gi|3789825 atCGcCTggGatcaaAgCCcgatgTTcaAGAaAcTGtATGAAGccgAA------------

gi|3896866 aCCGcCTggGaCcaGAgCCcgatcTTcaAGAaAATGtAcGAAGAggAA------------

gi|2956804 GCCGACcTTGTCGCGcACgATGCAcTTCAGgTAATGcgcGAAGcgcgAGCACGCGAACAG

gi|4017615 GCCGAtTTTGTCGCGcACgATGCAcTTCAGATAgTGggcaAAGcggcAGCAgGCGAACAG

gi|3898390 GCCGAtTTTaTCGCGAACaATGCATTTCAaATAATGcgcGAAacgcgAaCACGCaAACAt

gi|1676339 -------------------------------------ATGAAGAAAAg------------

gi|3796992 -------------------------------------ATGAAGAAAAg------------

gb|ABAM020 -------------------------------------ATGAAGAAAAg------------

gi|3927414 -------------------------------------ATGAAGAAAAg------------

gi|3635575 -------------------------------------ATGAAGAAAAg------------

gi|3536257 -------------------------------------ATGAAGAAAAg------------

gi|3751174 --------------------------------AgTGgAaaAAGAAgAA------------

gi|3789825 -TACGGCcAgttaGgtGgCgAACc-----------TTatGgCtGtATCaTtG-------C

gi|3896866 -TACGGCcAgttCGgcGgCgAgCc-----------gTtCGgCtGccTgGTAG-------g

gi|2956804 ATACGGaAgACGCGCcGAtAgAttCGCgTTCGCcGTTGCGTCCGgATCGTgGTACTCGaC

gi|4017615 gTACGGCAgACGaGagGcCAgACgCGCATTCGCaGTgGCaTCCGgATCGTgGTACTCGGC

gi|3898390 ATACGGtAAACGCGCtGAtAAgttCGCATTaGCgGTaGCGTCCGcATCaTAaTAtTCaGC

gi|1676339 ------------------------------------------------------------

gi|3796992 ------------------------------------------------------------

gb|ABAM020 ------------------------------------------------------------

gi|3927414 ------------------------------------------------------------

gi|3635575 ------------------------------------------------------------

gi|3536257 ------------------------------------------------------------

gi|3751174 ------------------------------------------------------------

gi|3789825 gGaTTaCTaCttCGACcataCaCCGcccgAtGTgGAtCTGCTtGGCtctATcGcCAaAGT

gi|3896866 CGaTTaCTaCttCGACcagtCGtCGccGgAcGTcGAgCTGCTcGGCGagcTgGcCAagGT

gi|2956804 CGGTTTCTGCAACGAtTGcGCaCCGATGAA-------------cGCcGCgTAGTCcGtaT

gi|4017615 CGGTTTtTGCAgaGAtTGcGCGCCaATGAA-------------GGCGGCAaAaTCAGAGT

gi|3898390 aGGcTTCTGtAAaGACTGtGCGCCGATaAA-------------GGCGGCATAGTCgGtGT

gi|1676339 ------------------------------------------------------------

gi|3796992 ------------------------------------------------------------

gb|ABAM020 ------------------------------------------------------------

gi|3927414 ------------------------------------------------------------

gi|3635575 ------------------------------------------------------------

gi|3536257 ------------------------------------------------------------

gi|3751174 ------------------------------------------------------------

gi|3789825 cgccGCGtcGgcCC----------AtgCGCcgTTtAttgccgggGctTCcCCCtCGGTac

gi|3896866 cTgcGCGGcG----------ATGcAtgCGCcgTTCAtCAgcgcgGcCTCGCCgACGGTga

gi|2956804 TcTTGCGGTGaAtgAGCGGTATGAAGCCaCTcTTggACAATTcCGctTCa-CggCGGTCt

gi|4017615 TTTTaCGGTGaACCAGCGGcATGAAGCCGtTtTTCgcCAgcTcgGctTCG-CggCGGTCg

gi|3898390 TTTTaCGaTGcACCAGCGGTATaAAGCCatTtTTCgcgAgTTcgGctTCG-CggCGaTCa

gi|1676339 --------------------------------------AATTTCGTCTCGCCCACGGTC-

gi|3796992 --------------------------------------AATTTCGTCTCGCCCACGGTC-

gb|ABAM020 --------------------------------------AATTTCGTCTCGCCCACGGTC-

gi|3927414 --------------------------------------AATTTCGTCTCGCCCACGGTC-

gi|3635575 --------------------------------------AATTTCGTCTCGCCCACGGTC-

gi|3536257 --------------------------------------AATTTCGTCTCGCCCACGGTC-

gi|3751174 -----------------------------------AAaAATcTCGTCcCGCtCACttTC-

gi|3789825 tgcAaAtgGAcTcCTGgCaGGaACTGgCgAAtCCcCGCGAcCtgAcCAAaatCGtCAcCc

gi|3896866 tgGgcAtgGgcTcCTGgCaGGagCTGtCcAAcCCgCGCGAcCtgAcCAAgatCttCAcCA

gi|2956804 gAGATcGCGATTTCcGTCGGGCACT---------------TgATgTCgAtGCCGCCgTCA

gi|4017615 ctGATgGCGATcTCgGTCGGGCAtT---------------TCATgTCcAcGCCGCCgTCA

gi|3898390 gAaATAGCGATTTCTGTCGGGCAtT---------------TCATATCAAtGCCaCCATCA

gi|1676339 ------------------------------------------------------------

gi|3796992 ------------------------------------------------------------

gb|ABAM020 ------------------------------------------------------------

gi|3927414 ------------------------------------------------------------

gi|3635575 ------------------------------------------------------------

gi|3536257 ------------------------------------------------------------

gi|3751174 ------------------------------------------------------------

gi|3789825 agaacCtggAAtaTGcGccgtGGaaCTCGCTGCGgGcTagCGAaGAcTcCCgtTAtAttg

gi|3896866 c---gCcggAAtacGctGcctGGCgCTCGCTGCGcGaTtcCGAGGATTcaCgCTACAtCg

gi|2956804 TCcgTtGggAAcGTGtGacACGGC------------------AGGtTTTCgACTACACCA

gi|4017615 TCgcTtGggAAGGTGtGGcACGGC------------------AGGtTTTCgACTgCcCCA

gi|3898390 TCggTCGggAAaGTatGacACGGC------------------AGcccTTCCACcACgCCt

gi|1676339 ----TCGTAAAGGTGGGGTACG--------------------------------------

gi|3796992 ----TCGTAAAGGTGGGGTACG--------------------------------------

gb|ABAM020 ----TCGTAAAGGTGGGGTACG--------------------------------------

gi|3927414 ----TCGTAAAGGTGGGGTACG--------------------------------------

gi|3635575 ----TCGTAAAGGTGGGGTACG--------------------------------------

gi|3536257 ----TCGTAAAGGTGGGGTACG--------------------------------------

gi|3751174 ----TCGTAAAGGTGGGaTACG--------------------------------------

gi|3789825 gC-CTgaCgAtGCCGCGttTtcttGccCgCCtGCCGTA-----------------TggcG

gi|3896866 gC-CTgaCCAtGCCGCGttTCctcGccCgCCtGCCcTA-----------------cggcG

gi|2956804 CCGCTtTCCACGCCGCGAATCAgcGTGCACCAGCCGTAgTtcTTGAAcGAaCGGTTgATG

gi|4017615 CCGgacTCCACGCCGCGAATCgAgGTGCACCAGCCGTAcTCTTTGAAgGAGCGGTTgATG

gi|3898390 CCGCTtTCaACaCCGCGgATtAAtGTGCACCAGCCaTAtTCTTTGAAtGAGCGaTTgATG

gi|1676339 -------------------------------------------------------TAATG

gi|3796992 -------------------------------------------------------TAATG

gb|ABAM020 -------------------------------------------------------TAATG

gi|3927414 -------------------------------------------------------TAATG

gi|3635575 -------------------------------------------------------TAATG

gi|3536257 -------------------------------------------------------TAATG

gi|3751174 -------------------------------------------------------TAgTG

gi|3789825 caaAaACcaAcCcgGTgGaCGagtTTGattttGAAgaAGATgcggatGgttctGaCcata

gi|3896866 ccaAgACcgAcCcgGTgGatGcCtTcGcCttcGAggaAGAaAccAacGgCgcCGaCagCt

gi|2956804 tTGACcgccATCGCGTAcGCGGCGTTGGaCCAtAcATAGcattgATGaTCcGCGCCGTCG

gi|4017615 tTGgCcgccATCGCaTAGGCGGCGTTcGCCCAcgAATAGcTgttgTGGTttGCGCCGTCG

gi|3898390 tTaACggccATCGCGTAaGCaGCaTTaGCCCAGAcATActTAgaATGaTCcGCcCCGTCG

gi|1676339 ATGACACATATC------------------------------------------------

gi|3796992 ATGACACATATC------------------------------------------------

gb|ABAM020 ATGACACATATC------------------------------------------------

gi|3927414 ATGACACATATC------------------------------------------------

gi|3635575 ATGACACATATC------------------------------------------------

gi|3536257 ATGACACATATC------------------------------------------------

gi|3751174 ATGgCACATATC------------------------------------------------

gi|3789825 ccaAaTaCgTCtggAgCAAcgCgGcCTACgcGATggGcGtaAAcatCAACCGtTCcTTCA

gi|3896866 ccaAgTaCacCtgggcCAActCgGcCTAtgcGATggcgGtGAAcatCAACCGcTCgTTCA

gi|2956804 GTCgccTCTTCgAAATtgAAttcGTCgACtGGATTcGTcttgAcGCCA------------

gi|4017615 GTCtgcTCTTCAAAATCAAAgCtGTCgACcGGgTTAGTGCGgATGCCA------------

gi|3898390 GTCgtTTCTTCAAAgTgAAActcaTCTACcGGATTAGTaCGAATaCCA------------

gi|1676339 ---------------------------------------CGAATGCCA------------

gi|3796992 ---------------------------------------CGAATGCCA------------

gb|ABAM020 ---------------------------------------CGAATGCCA------------

gi|3927414 ---------------------------------------CGAATGCCA------------

gi|3635575 ---------------------------------------CGAATGCCA------------

gi|3536257 ---------------------------------------CGAATGCCA------------

gi|3751174 ---------------------------------------CGgATGCCA------------

gi|3789825 AaCaCTACGGCtGGtGtaCgttGAttCGCGGtgTGGAatCAGGCgGtGcggtgGAaAATC

gi|3896866 AgCtCTAtGGCtGGtGCtCgcGGAttCGCGGCgTGGAgtCcGGCgGcGAggtgccGAAcC

gi|2956804 -----TACGGCAGaCGCGCaAGGAAaCGCGGCATcG--CaAGGCCGAtATAGCGcGcgTC

gi|4017615 -----TACGGCAGGCGCGaCAGGAAGCGCGGCATca--CCAGcCCcAGgTAGCGgGcATC

gi|3898390 -----TAgGGCAaaCGCGCCAGGAAGCGCGGCATGG--CaAGcCCaAtATAGCGAGAATC

gi|1676339 ------------------------------------------------------------

gi|3796992 ------------------------------------------------------------

gb|ABAM020 ------------------------------------------------------------

gi|3927414 ------------------------------------------------------------

gi|3635575 ------------------------------------------------------------

gi|3536257 ------------------------------------------------------------

gi|3751174 ------------------------------------------------------------

gi|3789825 TTCCctgcCAtaCCTTCCCgAcTGAcgAtggcGGCGtGgACatGAAATgCccGACcgAaA

gi|3896866 TgCCGAcgCACaCCTTCCCgAccGAcgACggtGGCGtcgACatGAAATgCccGACcgAaA

gi|2956804 TTCgGAactACGC-------AGcGAgttCCAaGGCGCGTACTCGAggTTtTGcgtAAAGA

gi|4017615 TTCaGAttCACGC-------AGTGAAcgCCAgGcCGCGTAtTCGgtATTCTGGA---AGA

gi|3898390 TTC-------CGCCagCCtcAGTGAAttCCAgGcgGCGTAtTCGAgATTCTGcgtAAAGA

gi|1676339 ------------------------------------------------------------

gi|3796992 ------------------------------------------------------------

gb|ABAM020 ------------------------------------------------------------

gi|3927414 ------------------------------------------------------------

gi|3635575 ------------------------------------------------------------

gi|3536257 ------------------------------------------------------------

gi|3751174 ------------------------------------------------------------

gi|3789825 TCgcCaTCtCtGACcGcCGCGAGGcTgAaCtgGC-----------------------gAa

gi|3896866 TCgcCaTCgCcGACcGgCGCGAGGcggAGCtgGC-----------------------gAa

gi|2956804 TCTTCGTCA-----GGTCGCGcGGGTTcGCgAGtTCCTGCCAcGAgTCCATCTGCAGCAC

gi|4017615 TtTTgGTCA-----GGTCGCGcGGGTTAGCcAGCTCCTGCCAgGAcTCCATCTGCAtCAC

gi|3898390 TtTTgGTCA-----GGTCGCGAGGGTTgGagAGCTCCTGCCAgGAtTCCATCTGCAGaAC

gi|1676339 ------------------------------------------------------------

gi|3796992 ------------------------------------------------------------

gb|ABAM020 ------------------------------------------------------------

gi|3927414 ------------------------------------------------------------

gi|3635575 ------------------------------------------------------------

gi|3536257 ------------------------------------------------------------

gi|3751174 ------------------------------------------------------------

gi|3789825 aAACGGTtttaTcCCGtTGATccACcGtAAaaaCtCAGActAtGCCgCCTTtAtCgGCGC

gi|3896866 GAACGGTttcaTGCCGcTGcTGcACaagAAGaaCaCcGAcctCGCCgCCTTcAtCgGCGC

gi|2956804 GgACGGcGCtGcGCCGgTaATGAACGGcgcaTGCGatGctGcCGCCACCTTggCaAtCGa

gi|4017615 GccCGGTGCgGTaCCGgTGATGAACGGacAGTGtGCcGcaGAgcCgAtgcgcgCCAtttC

gi|3898390 agAtGGcGacGcGCCGcTaATaAAaGGaAcGTGCGCAGAgGcCGCgACtTTgcCaAGtGa

gi|1676339 ------------------------------------------------------------

gi|3796992 ------------------------------------------------------------

gb|ABAM020 ------------------------------------------------------------

gi|3927414 ------------------------------------------------------------

gi|3635575 ------------------------------------------------------------

gi|3536257 ------------------------------------------------------------

gi|3751174 ------------------------------------------------------------

gi|3789825 a--CAGtcGCTgcAaAaaCcaCaGGAaTactACGAtCcgGAcGcgACggcCaACGctAAc

gi|3896866 G--CAatcGCTgcAgAagCcGgccGAGTaTGACGAcCcCGAcGccACcgcCaACGcCAAc

gi|2956804 GCCCAGCAGgTCgACAT-CgGCGGGtGTGTGA-----TCGAAGTAgtAgTCGgCGACgAg

gi|4017615 GCCCAGCAGCTCaACgTCCtGCGGGc-TGTGg-----TCGAAGTAgtAgTCGcCcACgAt

gi|3898390 agCCAGCAGaTCtACATCagGtGcGc-TGTGA-----TCaAAaTAAtAATCGgCaACcAg

gi|1676339 ------------------------------------------GTAACAAT----------

gi|3796992 ------------------------------------------GTAACAAT----------

gb|ABAM020 ------------------------------------------GTAACAAT----------

gi|3927414 ------------------------------------------GTAACAAT----------

gi|3635575 ------------------------------------------GTAACAAT----------

gi|3536257 ------------------------------------------GTAACAAT----------

gi|3751174 ------------------------------------------GTAACAAT----------

gi|3789825 CtgtCtgCcCGtCTacCgtaCCtGTTcgCCtgctcgCgCTTCGctcAcTTcCTcAAAtgt

gi|3896866 CtgGCggCACGcCTgcCCtaCCtGTTcgCCAcctgCCgCTTCGcccATTacCTgAAgtgc

gi|2956804 aCAGCCAtACGGCTCGCCaCCCAacTGCCCgTACTCtTCcTCGTAGATTTgCTTgAAgAa

gi|4017615 gCAGCCAaACGGCTCGCCgCCaAacTGaCCATACTCCTgcTCGTAGATTTtCTTgAAgAt

gi|3898390 gCAaCCAtAaGGCTCGCCcCCaAGTTGCCCATACTCtTCTTCGTAGATTTttTTAAAcAg

gi|1676339 ------------------------------------------------------------

gi|3796992 ------------------------------------------------------------

gb|ABAM020 ------------------------------------------------------------

gi|3927414 ------------------------------------------------------------

gi|3635575 ------------------------------------------------------------

gi|3536257 ------------------------------------------------------------

gi|3751174 ------------------------------------------------------------

gi|3789825 atcGtcCgcGaCaaAAtCGgttCCTTTAAAGaGCgTgAgGaTAtGCaGC-----------

gi|3896866 atcGtTCgcGaCaagAtCGgCtCgTTcAAgGaaaaggAcGacAtGCaGC-----------

gi|2956804 CGGGCTCTGGTCCCAcgCaAtcCCTTTAtAGCGCTTCAttGTgCGCCGCAGgTCgTctTT

gi|4017615 CGGGCTCTGGTCCCAgcCcACgCCcTTgAAGCGCTTCAgGGTACGgCcCAGcTCcTgCTT

gi|3898390 CGGGCTtTGaTCCCAggCGACgCCTTTAtAGCGtTTCAtcGTACGgCGCAGgTCactCTT

gi|1676339 ------------------------------------------------------------

gi|3796992 ------------------------------------------------------------

gb|ABAM020 ------------------------------------------------------------

gi|3927414 ------------------------------------------------------------

gi|3635575 ------------------------------------------------------------

gi|3536257 ------------------------------------------------------------

gi|3751174 ------------------------------------------------------------

gi|3789825 --gCtgGCTaAAtgAatGGATTaTgAAtTat-------------------------GtcG

gi|3896866 --gCtgGCTgcAGAActGGATTcTCAAtTaC-------------------------GtgG

gi|2956804 cGACAcatcCAtGAAGCGGATcTTCAgCTTCTCaTCCGTTTCcGTGTTcGcgACgAGGTG

gi|4017615 gGAgAtGCTCAtaAAGCGGATcTTCAgCaTCTCGTCaGTTTCgGTGTTgtTcACcAGGTa

gi|3898390 gGAaAtatcCAgGAAGCGtAaTTTtAACTTtTCGTCCGTTTCgGTaTgaGTgACgAGaTa

gi|1676339 ------------------------------------------------------------

gi|3796992 ------------------------------------------------------------

gb|ABAM020 ------------------------------------------------------------

gi|3927414 ------------------------------------------------------------

gi|3635575 ------------------------------------------------------------

gi|3536257 ------------------------------------------------------------

gi|3751174 ------------------------------------------------------------

gi|3789825 ACGCcGAtCCgGtgAActcCTCgCAAGaaaCtaAAGCccgTcgTCCGcTGGctGccgctG

gi|3896866 ACGgcGACCCgGCCcActcCaCcgAAaCcaCCaAgGCccagcAcCCctTGGccGcggcGG

gi|2956804 AtGaAGtCCaCGCCAcGCGCTtTCAAGactCtGAAagTcTTcAT--GgTGGAgaATCAGa

gi|4017615 gCtCAGACCgCGCCAgGCGCTCTCcAGCttCtGAAaCTcTTcgT--GgTGaAtGATCtGG

gi|3898390 AtGCAGACCgCGCCAgGCGCTtTCcAGCgtCtGAAaCTccTcAT--GaTGtAaaATCAGG

gi|1676339 -----------------------------GCCGAAGCTTTTTAT----------------

gi|3796992 -----------------------------GCCGAAGCTTTTTAT----------------

gb|ABAM020 -----------------------------GCCGAAGCTTTTTAT----------------

gi|3927414 -----------------------------GCCGAAGCTTTTTAT----------------

gi|3635575 -----------------------------GCCGAAGCTTTTTAT----------------

gi|3536257 -----------------------------GCCGAAGCTTTTTAT----------------

gi|3751174 -----------------------------GCCGAAGCTTTTTAT----------------

gi|3789825 aaGtagTGgTgGaAGAGgTcGAaGgCAATCCaGGttATtAcGacgcGAA-----------

gi|3896866 agGtggTGgTCGaAGAGgTcGAgGgCAATCCCGGttActAcaGctcGAA-----------

gi|2956804 TTGAtcTGtTCGGAtAatTtGcgaTCgATCtCccCAATaATcGActctATGtTTTTATAt

gi|4017615 TTcAccTGcTgaGAGAGcTgttcGTCgATaCCGGCAATCAgGttctGAATGgTgcgATAg

gi|3898390 TTGAttTGcTCtGAaAGcTttctGTCAATttCaGCAATgATGGAtgcAATGcaTTTATAa

gi|1676339 ------------------------------------ATCAT-------------------

gi|3796992 ------------------------------------ATCAT-------------------

gb|ABAM020 ------------------------------------ATCAT-------------------

gi|3927414 ------------------------------------ATCAT-------------------

gi|3635575 ------------------------------------ATCAT-------------------

gi|3536257 ------------------------------------ATCAT-------------------

gi|3751174 ------------------------------------ATtAT-------------------

gi|3789825 -------------------------------------atTCTtCCtGCGTCCGcAtttCc

gi|3896866 -------------------------------------GtTCTaCCtGCGcCCGcAttaCc

gi|2956804 GCgTCGTCgGAcAcCGTtgCcGaggTAAgCAGCGCCTGCTgaGCgAGCGTCCGGACaGCt

gi|4017615 GtATCGTtaGAgAagGTAACgGtATTctCCAGCGCCTGCTgTGCCAGgGTCttGACCGCg

gi|3898390 GCATCaTCcGAgAgCGTAACaGcATTAACCAGtGCCTGCTCcGCaAGgGTtttGACCGCt

gi|1676339 ------------------------------------------------------------

gi|3796992 ------------------------------------------------------------

gb|ABAM020 ------------------------------------------------------------

gi|3927414 ------------------------------------------------------------

gi|3635575 ------------------------------------------------------------

gi|3536257 ------------------------------------------------------------

gi|3751174 ------------------------------------------------------------

gi|3789825 agCTtGAaGGgcTgACGgGATCGCTgCGcCTGGtGaCaAAACTgCCGtcaGtGaAGCagG

gi|3896866 agCTCGAaGGgcTCACGgtATCGCTgCGgCTGGtatCcAAgCTtCCatccGccaAGgcgG

gi|2956804 CcCTCGACaGCtTCgCGCGcgCGCTCCGttTtGGGcttgAAtTCgCGcgacAGgAGtGCG

gi|4017615 CttTCaACGGCAgaACGCGcctGaTCgGttTtcGGgCggAACTCtttgtTcAGcAGCGCG

gi|3898390 CctTCtACcGCAgCACGaGtTtGaTCCGaacGcGGtttAAACTCCttagcGAGTAaCGCG

gi|1676339 -------------------------------------------------TGAGTAG----

gi|3796992 -------------------------------------------------TGAGTAG----

gb|ABAM020 -------------------------------------------------TGAGTAG----

gi|3927414 -------------------------------------------------TGAGTAG----

gi|3635575 -------------------------------------------------TGAGTAG----

gi|3536257 -------------------------------------------------TGAGTAG----

gi|3751174 -------------------------------------------------TGAGTAG----

gi|3789825 gcaAtGcCtGA-------------------------------------------------

gi|3896866 TTtgA-------------------------------------------------------

gi|2956804 TTGAAaTCgGACTGaTT---GGtCacgCtCGCCGttgcCTtgGtgcGCgtAtTCgcttga

gi|4017615 cTGAAtTCAtcCTGgcTgAaGGCCtgaCCCGCCtgctgCTcatgTTGtTgAGTCtg----

gi|3898390 TTGAAGTCAtcagGtTcaAtGGCagacgCCGCgGcgcgCTgtGaTTGCTctGT-------

gi|1676339 ------------------------------------------------------------

gi|3796992 ------------------------------------------------------------

gb|ABAM020 ------------------------------------------------------------

gi|3927414 ------------------------------------------------------------

gi|3635575 ------------------------------------------------------------

gi|3536257 ------------------------------------------------------------

gi|3751174 ------------------------------------------------------------

gi|3789825 ------------------------------------------------------------

gi|3896866 ------------------------------------------------------------

gi|2956804 agttcggccatcatttcccccatcgtttcaggctgtgctttcgtgccgaaagctaaAtTC

gi|4017615 -----------------------------------------------------GTtgcTC

gi|3898390 -----------------------------------------------------GTgAgTC

gi|1676339 ------------------------------------------------------------

gi|3796992 ------------------------------------------------------------

gb|ABAM020 ------------------------------------------------------------

gi|3927414 ------------------------------------------------------------

gi|3635575 ------------------------------------------------------------

gi|3536257 ------------------------------------------------------------

gi|3751174 ------------------------------------------------------------

gi|3789825 --

gi|3896866 --

gi|2956804 AT

gi|4017615 AT

gi|3898390 AT

gi|1676339 --

gi|3796992 --

gb|ABAM020 --

gi|3927414 --

gi|3635575 --

gi|3536257 --

gi|3751174 --

*Legend:
Similar residues are colored as the most conserved one (according to BLOSUM62).
Average BLOSUM62 score: Max: 3.0 Mid: 1.5 Low: 0.5*

Figure S1C

gi|1676339 -----TTACTTGTTGAGTGAATTcGAATCCGGGGGGGCCAGAAGTAGCAgAgAAAAGGTt

gi|3789874 -----TTACTTGTTGAGTGAATTcGAATCCGGGGGGGCCAGAAGTAGCAgAgAAAAGGTt

gi|1972615 -----TTACTTGTTGAGTGAATTcGAATCCGGGGGGGCCAGAAGTAGCAgAgAAAAGGTt

gi|3876104 -----cTAtTgtTTtgaTGAATcAGtA------GGtGCaAGcAtTAGCAtACtgAAaGTG

gi|2388994 -----cTAtTgtTTtgaTGAATcAGtA------GGtGCaAGcAtTAGCAtACtgAAaGTG

gi|1675899 atgccTccCcTtgTaAGgGgggTAGcATattGtcatGCtAacgacgtaAccCAAcAtaTG

gi|3884761 ------------------------------------------------------------

gi|1676339 GcAAAATAcCAgATtAGAAAAAgAGgCATACCAAAGCCatcGCAAGCgAAATCTTCAGCA

gi|3789874 GcAAAATAcCAgATtAGAAAAAgAGgCATACCAAAGCCatcGCAAGCgAAATCTTCAGCA

gi|1972615 GcAAAATAcCAgATtAGAAAAAgAGgCATACCAAAGCCatcGCAAGCgAAATCTTCAGCA

gi|3876104 GAAAAATAACAAATcAaAAAAATcaTCgaACCAttGCCTGAaCAgGCAAAATCTTCgGCt

gi|2388994 GAAAAATAACAAATcAaAAAAATcaTCgaACCAttGCCTGAaCAgGCAAAATCTTCgGCt

gi|1675899 GAtgtgaAAttAATgttAAgtgTttTtATtCCttcatCTGAG--AGatgtgTtTcCAGat

gi|3884761 ------------ATgttAcgtATAtTtATcCCAAcatCTaAc--gGtAAgATtTcCAGac

gi|1676339 AcCATTAT--------------CAcGAagGAGgcAATATAgAt--------------TAC

gi|3789874 AcCATTAT--------------CAcGAagGAGgcAATATAgAt--------------TAC

gi|1972615 AcCATTAT--------------CAcGAagGAGgcAATATAgAt--------------TAC

gi|3876104 ATCATTgT--------------gATGATAGAGATgATATATAC--------------TgC

gi|2388994 ATCATTgT--------------gATGATAGAGATgATATATAC--------------TgC

gi|1675899 gTCgTTATtTATTaTCATTcgcgcTaATAaAtATAATATtTtCCaTTtTggttggggTtt

gi|3884761 gTCgTTATaTATTtTCATTtatttTaATAaAttTcATtTtTgCCtTTcT--------TAt

gi|1676339 aAGCGTACCCcAT-------ACATAGaATTTTATATAtTCAAAGCCACTgT-------Ct

gi|3789874 aAGCGTACCCcAT-------ACATAGaATTTTATATAtTCAAAGCCACTgT-------Ct

gi|1972615 aAGCGTACCCcAT-------ACATAGaATTTTATATAtTCAAAGCCACTgT-------Ct

gi|3876104 TAatGTACCaAAa-------ACATAaGtTTTTATATAgatgAAaCCACTAT-------CA

gi|2388994 TAatGTACCaAAa-------ACATAaGtTTTTATATAgatgAAaCCACTAT-------CA

gi|1675899 TAttaTAtttAAgctTtGtGAtActGGcaaTTtTATtcaCAAttttACTA--------CA

gi|3884761 TAtCtTttttAAcgaTgGaGAagcAGGcTTTctTgTtaTagtctCtACcATcgtactcCA

gi|1676339 CTGAATCGCTGACAgTTTAAATTaATGACGAGATAATG--------TAGTAaAATTGTgA

gi|3789874 CTGAATCGCTGACAgTTTAAATTaATGACGAGATAATG--------TAGTAaAATTGTgA

gi|1972615 CTGAATCGCTGACAgTTTAAATTaATGACGAGATAATG--------TAGTAaAATTGTgA

gi|3876104 CgGAgTCGCTGgCAATTcAtgTTGATGACGAGATAATGGAGTACGATgGTAGAgactatA

gi|2388994 CgGAgTCGCTGgCAATTcAtgTTGATGACGAGATAATGGAGTACGATgGTAGAgactatA

gi|1675899 tTatcTCGtcattAATTTAAAcTGtcagCGAttcAgaG-------AcAGTgGctTTGaat

gi|3884761 tTatcTCGtcatCAAcaTgAATTGccagCGActccgTG-------ATAGTgGttTcaTct

gi|1676339 ATAAAAttGCCaGTATCACAAAGCTTAAATATAATAAAACCCCAACCAAAATGGAAAATA

gi|3789874 ATAAAAttGCCaGTATCACAAAGCTTAAATATAATAAAACCCCAACCAAAATGGAAAATA

gi|1972615 ATAAAAttGCCaGTATCACAAAGCTTAAATATAATAAAACCCCAACCAAAATGGAAAATA

gi|3876104 AcAAgAAAGCCTGctTCtCcAtcgTTAAAaAagATAAtA--------AgAAaGGcAAAaA

gi|2388994 AcAAgAAAGCCTGctTCtCcAtcgTTAAAaAagATAAtA--------AgAAaGGcAAAaA

gi|1675899 ATAtAAAAttCTaTgT-------aTggggTAcgcTtgtA--------------atctATA

gi|3884761 ATAtAAAAaCtTaTgT-------tTTtggTAcAtTAgcA--------------GtAtATA

gi|1676339 TTATATTTATTAGCGCGAATGATAATAAATAACGACATCTGGAAAcAcATCT--CTCAGA

gi|3789874 TTATATTTATTAGCGCGAATGATAATAAATAACGACATCTGGAAAcAcATCT--CTCAGA

gi|1972615 TTATATTTATTAGCGCGAATGATAATAAATAACGACATCTGGAAAcAcATCT--CTCAGA

gi|3876104 TgAaATTTATTAaaataAATGAaAATAtATAACGACgTCTGGAAATcTtaCc--gTtAGA

gi|2388994 TgAaATTTATTAaaataAATGAaAATAtATAACGACgTCTGGAAATcTtaCc--gTtAGA

gi|1675899 TTgccTcctTcgtg--------------ATAAtGgttgCTGaAgATtTcgCTTGCgatGg

gi|3884761 TcATcTcTATcAtC--------------AcAAtGAtAgCcGaAgATtTtgCcTGtTCAGg

gi|1676339 TGAaGGAATAAAAAcACTTAACATTAATTTCACATCCATATGTTGGGTTACGTCGTTAGC

gi|3789874 TGAaGGAATAAAAAcACTTAACATTAATTTCACATCCATATGTTGGGTTACGTCGTTAGC

gi|1972615 TGAaGGAATAAAAAcACTTAACATTAATTTCACATCCATATGTTGGGTTACGTCGTTAGC

gi|3876104 TGtTGGgATAAAtATACgTAACAT------------------------------------

gi|2388994 TGtTGGgATAAAtATACgTAACAT------------------------------------

gi|1675899 cttTGGtATgcctcTttTTctaATctggTattttgCaAccTtTTctcTgctacttcTgGC

gi|3884761 caATGGttcgAtgATttTTttgATTtgTTattttTCCActTtcaGtaTgctaatGcTtGC

gi|1676339 ATGACAATATGCTACCCCCCTTACAAGGGGAGGCAT

gi|3789874 ATGACAATATGCTACCCCCCTTACAAGGGGAGGCAT

gi|1972615 ATGACAATATGCTACCCCCCTTACAAGGGGAGGCAT

gi|3876104 ------------------------------------

gi|2388994 ------------------------------------

gi|1675899 ccccCcggATtCgAattCaCTcAacAaGtaA-----

gi|3884761 Acc------TaCTgattCatcaAaAcaatag-----

*Legend:
Similar residues are colored as the most conserved one (according to BLOSUM62).
Average BLOSUM62 score: Max: 3.0 Mid: 1.5 Low: 0.5*
