# Supplementary material for: Genes ycfR, sirA and yigG Contribute to the Surface Attachment of Salmonella enterica Typhimurium and Saintpaul to Fresh Produce
Source: PLoS One. 2013 Feb 22;8(2):e57272. doi: 10.1371/journal.pone.0057272 (PMC3579871; doi:10.1371/journal.pone.0057272)
Supplement: Table S1 — Primers used in this study. A list of all primers used in the study along with sequences. (DOCX) [file pone.0057272.s002.docx]

**Table S1.** Primers used in this study.

| Primer name | Sequence (5’-3’) | Usage |
| --- | --- | --- |
| JKS-8 | CGCGAGGCTTTTTCTTTTTATTG  TGGCAATCGCAGCGGCATTAAT  GAGGGTTAATGCTGgagctgcttcga  gttcctatac | *ycfR* knock-out primer including *ycfR* upstream homology arm (capitalized) and anti-sense primer for amplification of *cm* cartridge |
| JKS-9 | AACTTATCGATCGTTAAGTTAA  TGCTTACGACACCCATTCATCTG  CTAAAGGTCATCACTccatggtccat  atgaatatcctc | *ycfR* knock-out primer, including *ycfR* downstream homology arm (capitalized) and sense primer for amplification of cm cartridge |
| JKS-16 | CCATTTTTCAGATAGCGAACCAT  CATTAATATAATCCTTGTCCATGT  AAACTCCATTAAAgagctgcttcgaagtt  cctatac | *yigG* knock-out primer including *yigG* upstream homology arm (capitalized) and anti-sense primer for amplification of *cm* cartridge |
| JKS-17 | CGATTTATACGCAGCGTAAAAA  ATAACGCGACTATTATTCTAAAT  ATAATCTGAAAAAATccatggtccat  atgaatatcctc | *yigG* knock-out primer, including *yigG* downstream homology arm (capitalized) and sense primer for amplification of cm cartridge |
| JKS-18 | TATATTTTGTGAATGTTTAAGC  GAGTGAAGTCAGAGAAGATAGA  GAATATAAAGAGGGATgagctgcttc  gaagttcctatac | *sirA* knock-out primer including *sirA* upstream homology arm (capitalized) and anti-sense primer for amplification of *cm* cartridge |
| JKS-19 | GAGCAGAACAATACCAGGTAA  CAGATGAAAAGTTGGGCTTAT  GGTCATAATATGTATTTCccatgg  tccatatgaatatcctc | *sirA* knock-out primer, including *sirA* downstream homology arm (capitalized) and sense primer for amplification of cm cartridge |
| KD-4 | ATGTCCGCCGTCATCAAGTG | Sense PCR primer for amplification of *ycfR* knockout region |
| KD-5-280 | CACCAGTCCAGATCCCATAA | Anti-sense PCR primer for amplification of *ycfR* knockout region |
| JKS-24 | TTGACGCCGACATAAGCTGC | Sense PCR primer for amplification of *yigG* knockout region |
| JKS-25 | CGCGTCTGCGTCTTTCTAGA | Anti-sense PCR primer for amplification of *yigG* knockout region |
| JKS-20 | GGATATGCAGCGCTGGCTAA | Sense PCR primer for amplification of *sirA* knockout region |
| JKS-21 | GTCTTCTGGCGATCCCTCAA | Anti-sense PCR primer for amplification of *sirA* knockout region |
| ycfR-F-comp | atGGATCCcatagagagtaggcgctttc | Forward primer to clone *ycfR* gene and its 400 bp promoter region with BamHI site (capitalized) |
| ycfR-R-comp | atGGATCCatcgcagcggcattaatgag | Reverse primer to clone *ycfR* gene and its 400 bp promoter region with BamHI site (capitalized) |
| yigG-F-comp | atGGATCCtccttgtccatgtaaactcc | Forward primer to clone *yigG* gene and its 400 bp promoter region with BamHI site (capitalized) |
| yigG-R-comp | atGGATCCgaagcgcagacgggtatgct | Reverse primer to clone *yigG* gene and its 400 bp promoter region with BamHI site (capitalized) |
| sirA-F-comp | atGGATCCgcttcgctcacttcctcaaa | Forward primer to clone *sirA* gene and its 400 bp promoter region with BamHI site (capitalized) |
| sirA-R-comp | atGGATCCagttgggcttatggtcataa | Reverse primer to clone *sirA* gene and its 400 bp promoter region with BamHI site (capitalized) |
